# Dibasic 3,3′-di­nitro-4,4′-bipyrazole-1,1′-diides of K, Rb and Cs reveal metal-selective ring–π over ring–N coordination

**DOI:** 10.1107/S2056989026005037

**Published:** 2026-05-19

**Authors:** Kostiantyn V. Domasevitch, Vira V. Ponomarova

**Affiliations:** a Inorganic Chemistry Department National Taras Shevchenko University of Kyiv Volodymyrska Str 64/13 01601 Kyiv Ukraine; University of Aberdeen, United Kingdom

**Keywords:** crystal structure, potassium, rubidium, caesium, nitro­pyrazoles, ionic pyrazolates, cation–π inter­action

## Abstract

Three new alkali metal pyrazolate salts, namely, poly[[μ-3,3′-di­nitro-4,4′-bipyrazole-1,1′-diido]dipotassium], [K_2_(C_6_H_2_N_6_O_4_)]_*n*_, (**1**), its rubidium analogue [Rb_2_(C_6_H_2_N_6_O_4_)]_*n*_, (**2**), and poly[[[μ-3,3′-di­nitro-4,4′-bipyrazole-1,1′-diido]dicaesium] monohydrate], {[Cs_2_(C_6_H_2_N_6_O_4_)]·H_2_O}_*n*_, (**3**), suggest that the electron-depleted nitro­pyrazolates may be still functional as efficient π-donors toward late alkali metal ions. The three-dimensional structures of **1** and **2** are very similar, being governed by μ_4_-κ^1^:κ^1^:κ^1^:κ^1^ coordination of the pyrazolate anions as hard Lewis bases [K—N = 2.783 (3)–2.956 (3); Rb—N = 2.930 (4)–3.194 (4) Å]. In contrast, the structure of **3** reveals a dominant significance for the pyrazolate–π coordination, which produces two distinct motifs in the forms of finite Cs—π arrangement [Cs—ring centroid distance = 3.389 (3) Å] and one-dimensional —Cs—(π—Cs)_*n*_— sandwiches with slightly larger Cs—π separations at 3.474 (3) and 3.587 (3) Å. Weaker ion–dipole inter­actions with the nitro-O donors, at the distances approaching the sums of corresponding ionic radii, are relevant for the entire series and they complete typically high coordination numbers of the metal ions [CN = 8, 9]. These findings may find use in the construction of ion-selective receptors since the existing examples for the coordinative discrimination of the late alkali metal ions, beyond the ion-cavity size match considerations, are still rare.

## Abstract 2

A series of late alkali metal salts introduce electron-depleted nitro­pyrazolate anions as common N-ligands for K^+^ and Rb^+^, but π-ligands for softer Lewis acid Cs^+^.

## 1. Chemical context

The crystal chemistry of alkali metals azolates (deprotonated five-membered aromatic nitro­gen heterocycles with one or more N atoms) provides appropriate models and paradigms for assessing the significance of cation–π inter­actions, which are energetically superior to the other kinds of aromatic bonding and are applicable for control of solid-state architecture (Yamada, 2020 ▸). The cation–π bonding essentially expands the relatively scarce coordination landscape of alkali metal ions (Fabbrizzi, 2020 ▸), being a valuable factor for differentiation of their coordination behavior and enabling the construction of artificial ion-selective receptors. The relevance of such inter­actions to biology is also well established, in particular for mediating high K^+^-selectivity of biological ion channels (Dougherty, 2025 ▸).

For the most favorable case of electron-rich pyrrolate species, the relatively strong cation–π inter­actions are likely less sensitive to the nature of cation and they are frequently observed for any of the alkali metals [Li to Cs; Blanco *et al.*, 2008 ▸]. That is contrary to polynitro­gen azolate anions, *e.g.* pyrazolates, whose propensity for π-bonding is often hidden in the shade of their more competitive inter­actions as efficient multiple N–σ donors. Theoretical studies of Li, Na and K pyrazolates reveal that *M*—N coordination is the most favorable. Although in the case of Na and K two alternative structures are closer in stability (Cortés-Llamas *et al.*, 2006 ▸), all *M*–π-pyrazolate configurations spontaneously revert to the more stable N–σ ones (Blanco *et al.*, 2008 ▸). This may be attributed not only to the increased number of such donor-N sites, but also to the essential decrease in π-electron density upon the accumulation of endocyclic N atoms. A similar impact for bonding preferences comes from the incorporation of an appropriately strong acceptor. In this way, the computational models of Na^+^-nitro­benzene pairs suggest a total destabilization of the configurations involving Na^+^ at the π-cloud (Watt *et al.*, 2009 ▸). The combination of these co-aligned factors in the case of nitro­pyrazolates evidently mitigates against π-coordination, but such destructive impact could be fatal primarily for the harder Lewis acids (Li^+^, Na^+^, K^+^). The late alkali metal ions are prone to support contacts with delocalized and diffuse electron densities over coordinating the centers of highest charge and therefore, in this case, the *M*–π coordination may be more tolerant to the π-electron depletion. One can postulate essential selectivity for *M*–π over *M*–σ-N coordination, depending on the Lewis hardness/softness of the metal ion. For example, while the crystal chemistry of Na and K phenolates is dominated by *M*—O coordination, Cs compounds exhibit more complex, if not a completely different, behavior with only a few Cs—O inter­actions accompanying the multiple π-coordination patterns (Pink & Sieler, 2007 ▸).

Keeping in mind these inputs, we have examined the K^+^, Rb^+^ and Cs^+^ salts of 3,3′-di­nitro-4,4′-bi­pyrazole (**1**–**3**, respectively) and report their structures here.
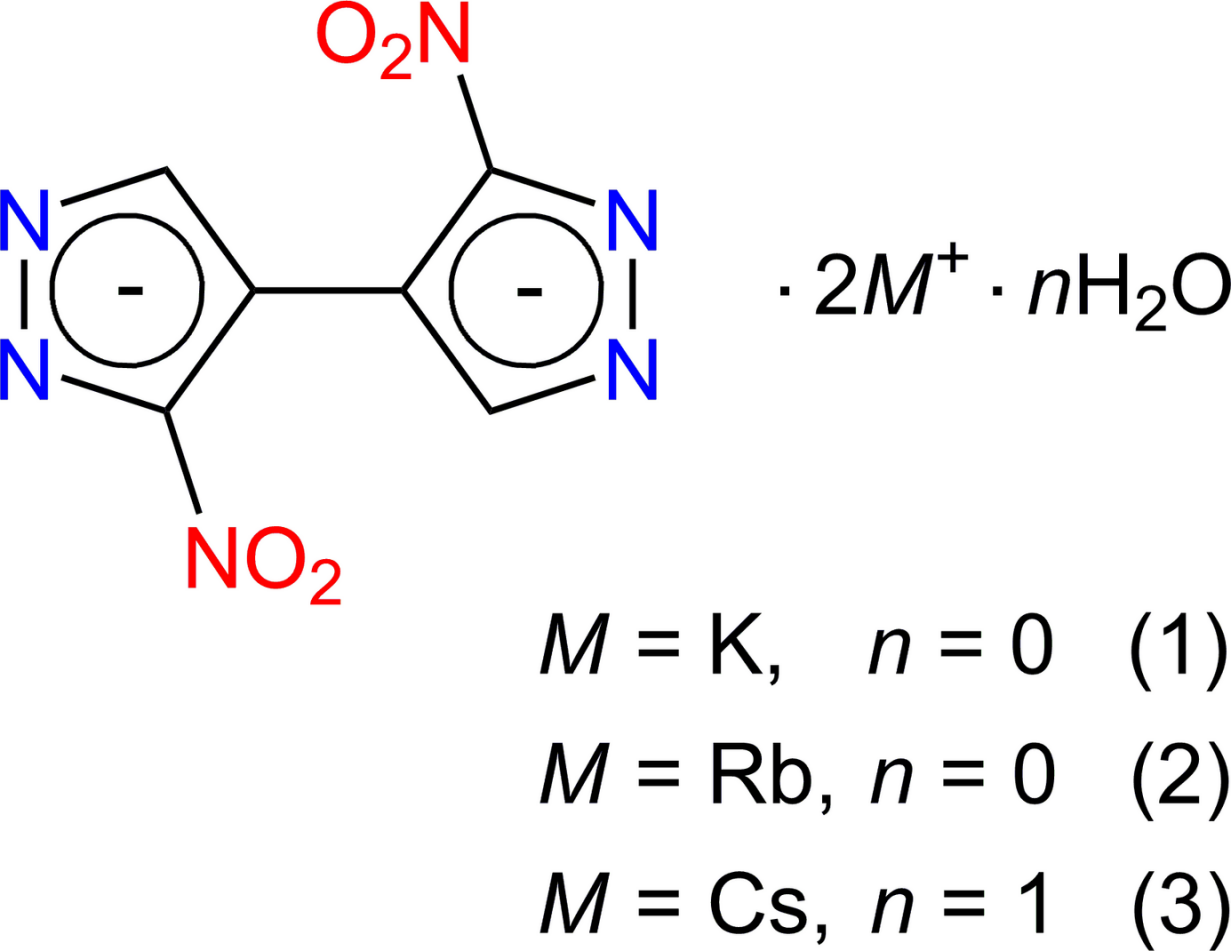


## 2. Structural commentary

**Figure 1:**
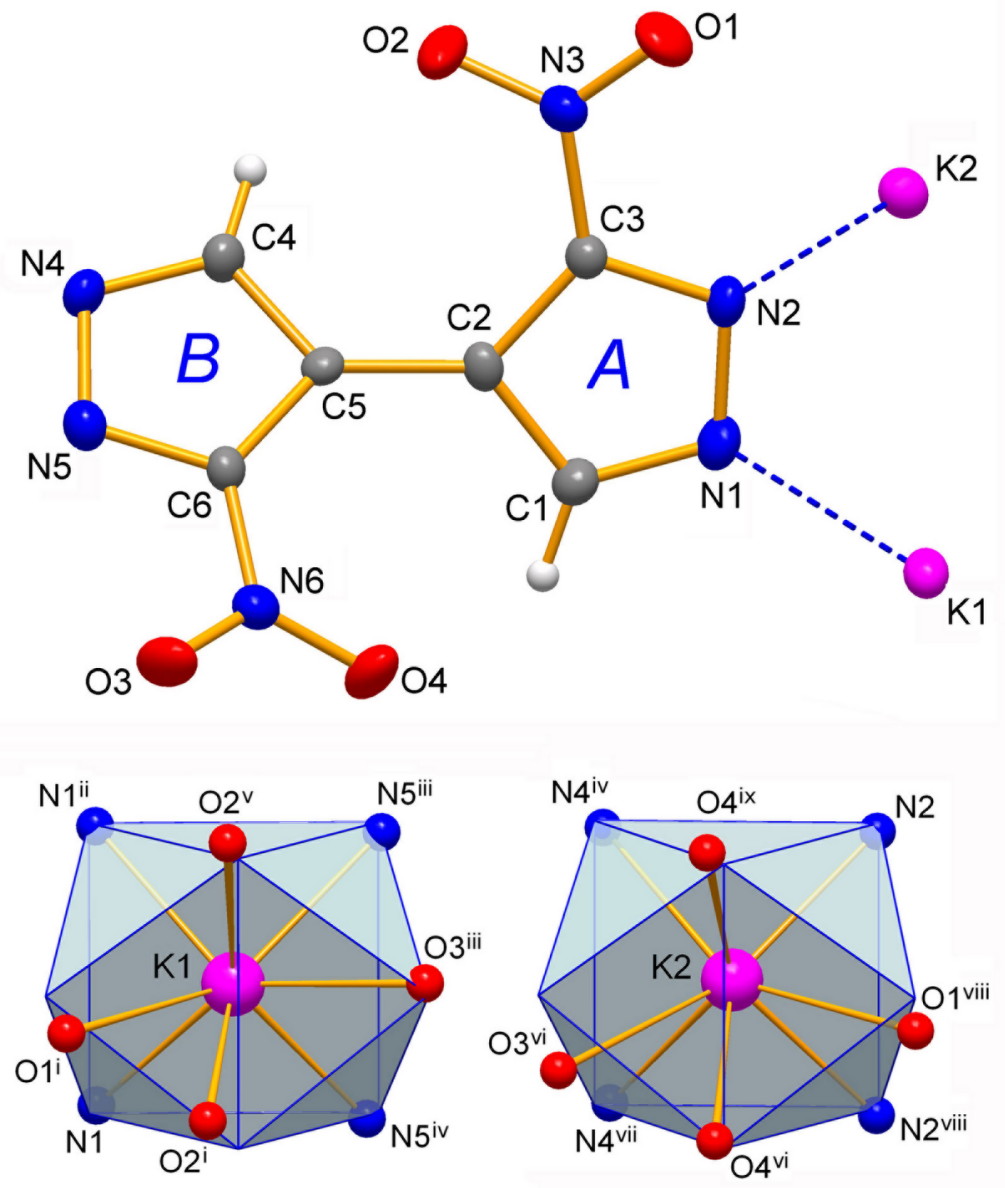
The mol­ecular structure of **1** with displacement ellipsoids at the 50% probability level. The coordination environments of two K ions are drawn against the best fitted idealized polyhedra in the form of biaugmented trigonal prisms. For symmetry codes, see Table 1 ▸.

The mol­ecular structures of the title compounds **1**–**3** are shown in Figs. 1 ▸–3 ▸ ▸, respectively. They represent similar dibasic salts, with the metal ions and organic dianions found in 2:1 proportions, and accommodating also one solvate water mol­ecule in the case of **3** [*M* = Cs]. The ease of crystallization of such salts from alkaline aqueous solutions is governed by the appreciable acidity of nitro substituted pyrazoles, which are weak NH acids comparable to phenols [p*K*_a_ = 9.81 for 3(5)-nitro­pyrazole *versus* 14.63 for the parent pyrazole; Janssen *et al.*, 1973 ▸].

The bonding preferences of K^+^ and Rb^+^ ions are very similar. They adopt typically high coordinations with four pyrazolate-N atoms complemented by four (or five for Rb2 in **2**) nitro-O atoms at distances that approach the sums of the corresponding ionic radii for eight-coordinate environments [which are *M*—N = 2.97 and 3.07 Å; *M*—O = 2.89 and 2.99 Å for K and Rb ions, respectively; Shannon, 1976 ▸]. However, some of the *M*—O bonds are essentially elongated indicating the weakness of these relatively distal ion–dipole inter­actions (Tables 1 ▸ and 2 ▸). One can find their median lengths (K—O = 2.96 Å; Rb—O = 3.11 Å) slightly exceeding the above sums of ionic radii whereas the situation for the *M*—N bonding is the reverse (median values for K—N = 2.86 Å; Rb—N = 3.02 Å). The latter could be regarded as a perceptibly stronger and dominant inter­action in the structures, as is anti­cipated for the relatively hard Lewis acid (*M*^+^) and base (pyrazolate-N^−^) duo. For comparison, without contribution from *M*—O inter­actions in homoleptic [K(Me_2_pz)]_*n*_ [Me_2_pz is 3,5-di­methyl­pyrazolate] the lengths of the K—N bonds fall into the range of 2.79 (1)–2.98 (2) Å (Woods, 2016 ▸), which exactly matches the corresponding parameters for **1**. The polyhedral geometries of the K ions in **1** represent similar biaugmented trigonal prisms with the appropriate shape measures of 2.454 (K1) and 3.153 (K2) (Ruiz-Martínez *et al.*, 2008 ▸), whereas in the case of the Rb ion in **2**, these are close to a cube [Rb1, CN = 8] and spherical-relaxed capped cube [Rb2, CN = 9] (shape measures are 3.550 and 5.112, respectively) (Figs. 1 ▸ and 2 ▸).

**Figure 3:**
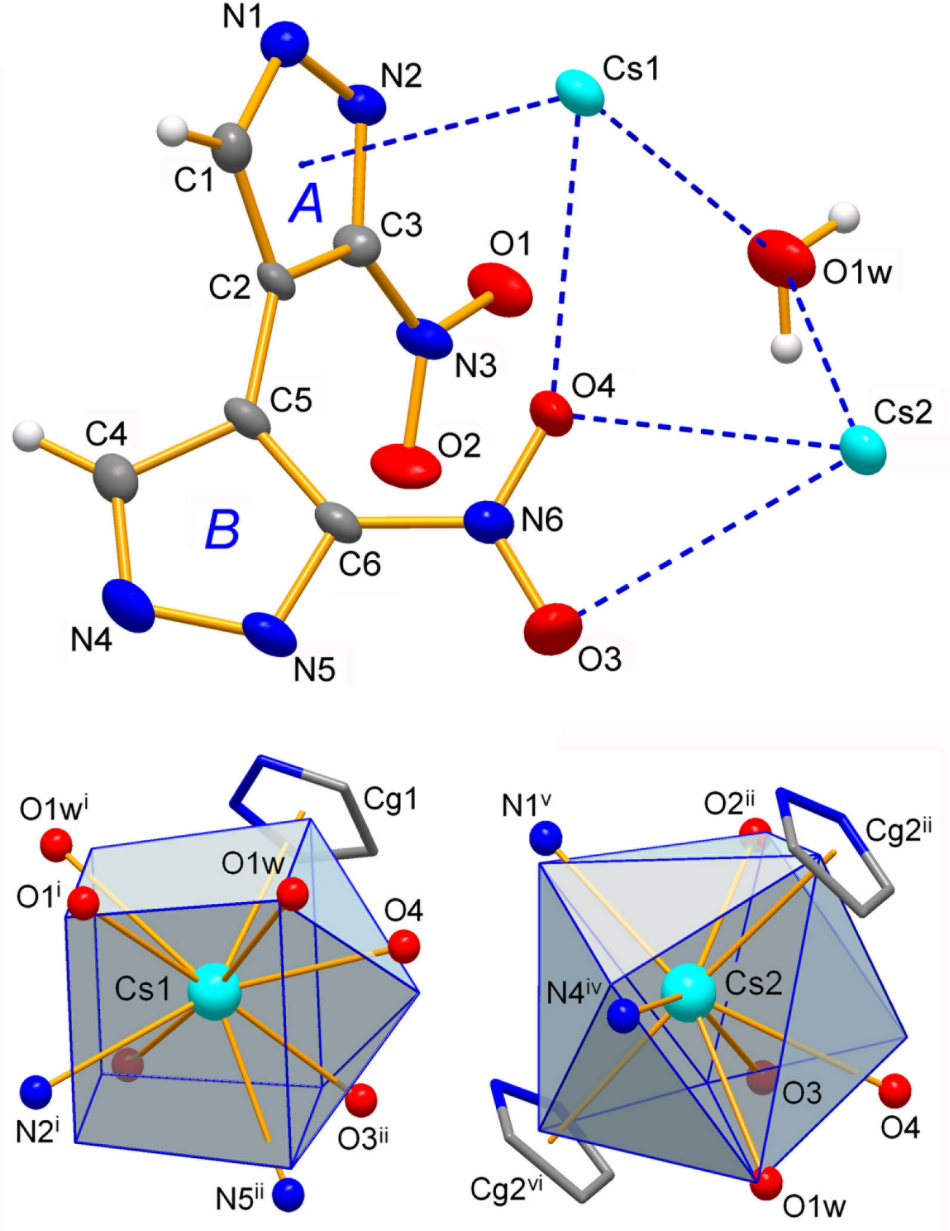
The mol­ecular structure of **3** with displacement ellipsoids at the 50% probability level. The coordination environments of two metal ions are drawn against the best fitted idealized polyhedra in the form of spherical-relaxed capped cube (Cs1) and triangular dodeca­hedron (Cs2) and when considering only the closest N1^v^ atom for κ^2^-coordinated pyrazolate group in the case of Cs2. For symmetry codes, see Table 3 ▸.

The bonding of the softer Lewis acid Cs^+^ is markedly different (Table 3 ▸) and these cations in **3** tend to reside at the π-clouds of the rings [Cs1—(ring *A*); Cs2—(ring *B*)^ii^ and Cs2—(ring *B*)^vi^; symmetry codes: (ii) −*x*, *y* − 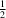, −*z* + 1; (vi) −*x*, *y* + 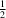, −*z* + 1] (Fig. 3 ▸), while supporting a set of relatively distal contacts with either N- or C-atoms over coordinating the centers of highest charge only, seen for **1** and **2**. Moreover, in the case of the Cs2—(ring *A*)^v^ pair, both Cs—N contacts are comparable in length [Cs2—N1^v^ = 3.251 (4) Å; Cs2—N2^v^ = 3.490 (4) Å; symmetry code (v) *x*, *y*, *z* − 1] and this suggests κ^2^-coordination. Such behavior results in partial elimination of Cs—N bonding in favor of new structure-directing inter­actions, namely cation–π bonds (Table 4 ▸). This parallels structural trends for *M*—π over *M*—N bonding in metallated porphyrinogens, which host Cs^+^ cations by fourfold π-coordination, whereas only two such inter­actions are actualized in the case of K^+^ cations due to their higher propensity to formation of more common K—N bonds (Bonomo *et al.*, 2001 ▸). In this way the ninefold coordination of Cs1 comprises one pyrazole-π donor, but even two π-ligands are bonded in the case of eight-coordinate Cs2. These environments are completed with two pyrazole-N [Cs—N = 3.132 (5)–3.392 (6) Å; median 3.26 Å]; nitro- and aqua-O atoms [Cs—O 3.064 (5)–3.754 (7) Å; median 3.17 Å] at the distances approaching the sums of corresponding ionic radii (Cs—N = 3.20; Cs—O = 3.12 Å; Shannon, 1976 ▸). The average bond length of eight-coordinate Cs with O-ligands is also in agreement (3.245 Å; Leclaire, 2008 ▸). The distorted coordination polyhedra of the Cs^+^ ions are nearly inter­mediate between several idealized geometries. The attribution of these configurations as spherical-relaxed capped cube [Cs1, CN = 9] and triangular dodeca­hedron [Cs2, CN = 8] is essentially nominal, while considering the best shape measure values of 7.035 and 10.076, respectively, and κ^2^-pyrazolate as only one vertex of the polyhedron for Cs2 (Ruiz-Martínez *et al.*, 2008 ▸). The relatively poor shape fits are in line with the distortions imposed by the combination of small κ^2^-NO_2_ and large η^5^-pyrazolate ligands.

Every local Cs-pyrazolate-π geometry features the metal ions situated almost exactly above the ring centroids, at the distances of 3.389 (3)–3.587 (3) Å (Table 4 ▸), and only slightly shifted from the centroid normal positions toward the ring-N atoms with the slippage angles of 7.5 (3)–20.1 (4)°. There are no precedents of Cs—π-pyrazolate bonding for direct comparison. Similar patterns within a pyrrolate (Heldt & Behrens, 2005 ▸; Bonomo *et al.*, 2001 ▸) and imidazolate series (Tadokoro *et al.*, 2001 ▸) suggest much stronger inter­actions with the Cs—π (centroid) distances as short as 3.069 Å. Nevertheless, the bonding in **3** may be regarded as very unusual and salient. The second inter­esting feature, which is unprecedented for nitro­pyrazolates, is double π-coordination. Unlike the finite arrangement of Cs1—π(ring *A*), the translation-related rings *B* and Cs2 ions sustain an infinite sandwich pattern. These Cs—π bonds are slightly weaker and less directional, in particular due to the elongation of Cs—C separations, up to 3.816 (6)–4.111 (5) Å. One can find that even the electron-depleted nitro­pyrazolate ligands retain the prominent ability for π-coordination and they are susceptible to inter­actions with the cations at both axial sides of the ring simultaneously. This kind of weakened bonding may be more selective for the softer Lewis acid Cs^+^, unlike stronger metal–π inter­actions with electron rich pyrazolates (Cortés-Llamas *et al.*, 2006 ▸).

The geometry of the organic dianions is consistent with the data for neutral 3,3′-di­nitro-4,4′-bi­pyrazole (Domasevitch *et al.*, 2019 ▸). Their common feature consists in a twisted conformation of the mol­ecular framework with two pyrazole rings rotated by 44.96 (7)–48.47 (15)°, while a slightly larger dihedral angle in the case of **3** [57.2 (2)°] is beneficial for specific bonding to Cs ions, which reside at the π-cloud of one ring and coordinate the nitro-O atom from the other ring (Fig. 3 ▸). Bond lengths within the pyrazole cores are also very similar to the neutral mol­ecule and its monofunctional prototype 3-nitro­pyrazole (Foces-Foces *et al.*, 1997 ▸). The most salient changes are associated rather with the angles at the ring N^1^- (C1—N1—N2 and C4—N4—N5) and N^2^-atoms (N1—N2—C3 and N4—N5—C6), which upon ionization become much closer in magnitude, namely 108.2 (4)–108.9 (4)° and 105.70 (17)–106.3 (4)°, respectively, *versus* the more significantly differentiated values of 113.52 (9) and 103.31 (9)° observed for the neutral mol­ecule (Domasevitch *et al.*, 2019 ▸). These ring angles are known as good signs for the protolytic state of 3,5-disubstituted pyrazoles in crystal structures since either pyrazolate or pyrazolium ions feature their exact equalization (Domasevitch, 2008 ▸). In the case of 3-nitro­pyrazolates the latter criteria may be less reliable as the anionic forms in **1**–**3** retain a perceptible difference of these parameters.

## 3. Supra­molecular features

**Figure 4:**
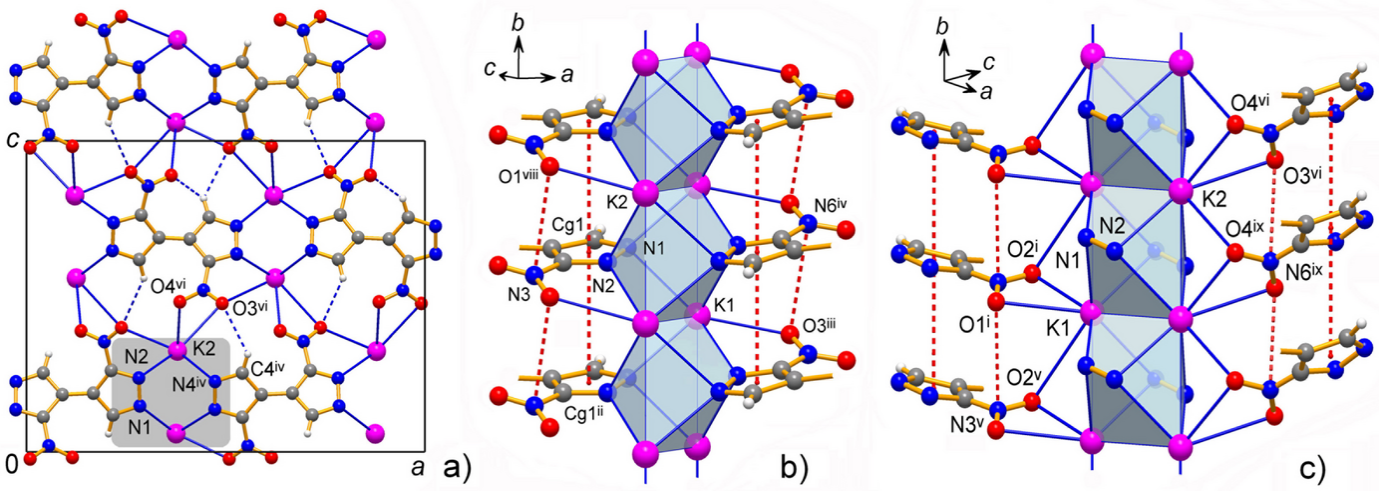
(*a*) Coordination layers in **1** viewed in the projection on the *ac* plane, with the grey box indicating primary K-pyrazolate chains, which are orthogonal to the drawing plane. (*b*) Side-view of the latter showing bridging function of pyrazolates and (*c*) bridging function of NO_2_ groups, with the particularly long K1⋯O2^v^ contacts. Red dotted lines indicate weak stacking inter­actions. [Symmetry codes: (i) −*x* + 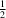, *y*, *z* − 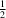; (ii) *x*, *y* − 1, *z*; (iii) *x* + 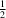, −*y* − 1, *z*; (iv) *x* + 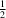, −*y*, *z*; (v) −*x* + 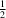, *y* − 1, *z* − 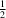; (vi) −*x* + 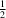, *y* + 1, *z* + 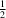; (viii) *x*, *y* + 1, *z*; (ix) −*x* + 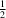, *y*, *z* + 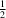.]

**Figure 5:**
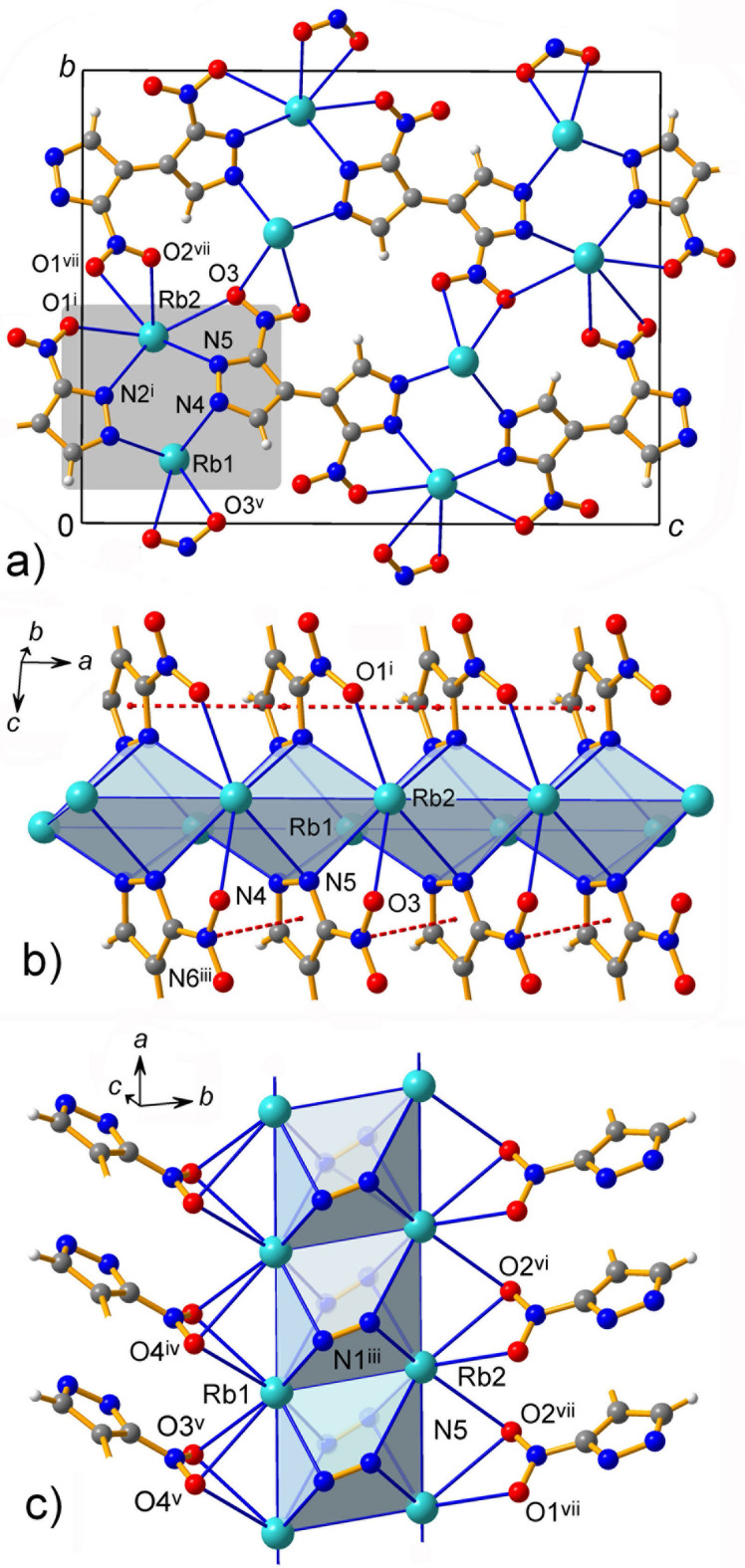
(*a*) Coordination layers in **2** viewed in the projection on the *bc* plane. The grey box indicates Rb–pyrazolate chains, which are orthogonal to the drawing plane. (*b*), (*c*) Two side views of the Rb–pyrazolate ladders along the *a*-axis direction showing the bridging function of the pyrazolates and NO_2_ groups. Red dotted lines indicate weak stacking inter­actions. [Symmetry codes: (i) *x* − 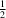, −*y* + 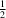, *z* − 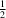; (iii) *x* + 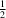, −*y* + 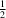, *z* − 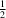; (iv) −*x* + 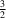, *y* − 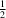, −*z* + 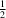; (v) −*x* + 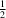, *y* − 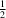, −*z* + 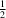; (vi) −*x* + 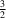, *y* + 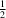, −*z* + 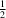; (vii) −*x* + 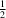, *y* + 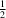, −*z* + 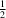.]

The extended structures of compounds **1** and **2** are very similar, both with regard to coordination and the resulting supra­molecular arrangements. The intrinsic significance of *M*—N coordination is reflected by the assembly of readily distinguishable tight metal-organic layers, which are further inter­connected in a third dimension with a set of weaker *M*–nitro-O inter­actions (Figs. 4 ▸ and 5 ▸). The primary *M*-pyrazolato connectivities themselves are only one-periodic and they are very simple. Double chains of metal ions adopt ladder-like motifs with the rung spacings 3.768 Å (**1**) and 3.967 Å (**2**) corresponding to the *b* and to the *a* parameters of the respective unit cells. Every section of these ladders accommodate two μ_4_-κ^1^:κ^1^:κ^1^:κ^1^ pyrazolate anions, above and below the *M*_4_ plane. This coordination mode is known, for example, for [Na_8_(*t*Bu_2_pz)_6_O] [*t*Bu_2_pz is 3,5-di(*t*-but­yl)pyrazolate; Beaini *et al.*, 2007 ▸], but it is relatively rare for alkali metal pyrazolates (Deacon *et al.*, 2000 ▸; Halcrow, 2009 ▸). A very subtle difference between these subconnectivities concerns the mutual orientation of the nitro groups, which are co-aligned in **2** (Fig. 5 ▸), but point in opposite directions in **1** (Fig. 4 ▸).

Since the organic anions are bifunctional, they serve as connectors between the adjacent coordination ladders that result in the generation of the layers, which are parallel to the *ab* plane in **1** and the *ac* plane in **2**. Accordingly, the inter­layer spacings correspond to ½*c* = 6.99 Å (**1**) or ½*b* = 7.10 Å (**2**) parameters of the unit cells. These separations are nearly identical and therefore one can suppose that the larger Rb^+^ ions support stronger inter­layer bonding. The latter concerns multiple *M*—nitro-O inter­actions and, indeed, they are more extensive in the case of *M* = Rb. In **1**, the coordination of NO_2_ is μ_3_-κ^2^:κ^1^:κ^1^ and three out of four K—O bonds are generated between the layers as the links for two adjacent K ions along the ladder. This chelate-bridging mode is frequently observed for alkali metal ions and nitro ligands (Mendoza-Báez *et al.*, 2024 ▸). However, one of the bonds per nitro group is significantly elongated [*e.g.* K1—O2^v^ = 3.418 (3) Å and K2—O3^vi^ = 3.153 (2) Å; symmetry codes: (v) −*x* + 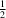, *y* − 1, *z -* 1/2; (vi) −*x* + 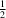, *y* + 1, *z* + 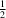] and they may be regarded as secondary weak dipole–dipole inter­actions. In **2**, the coordination modes are μ_3_-κ^2^:κ^1^:κ^1^ (N3/O1/O2) and μ_3_-κ^2^:κ^2^:κ^1^ (N6/O3/O4). The latter mode generates one additional M—O bond, as may be compared with the K analogue. In addition, even the most distal contact with the larger Rb ion [Rb2—O2^v^ = 3.370 (5) Å; symmetry code (v): −*x* + 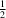, *y* − 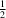, −*z* + 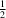] is shorter than the similar long bond for **1**.

**Figure 6:**
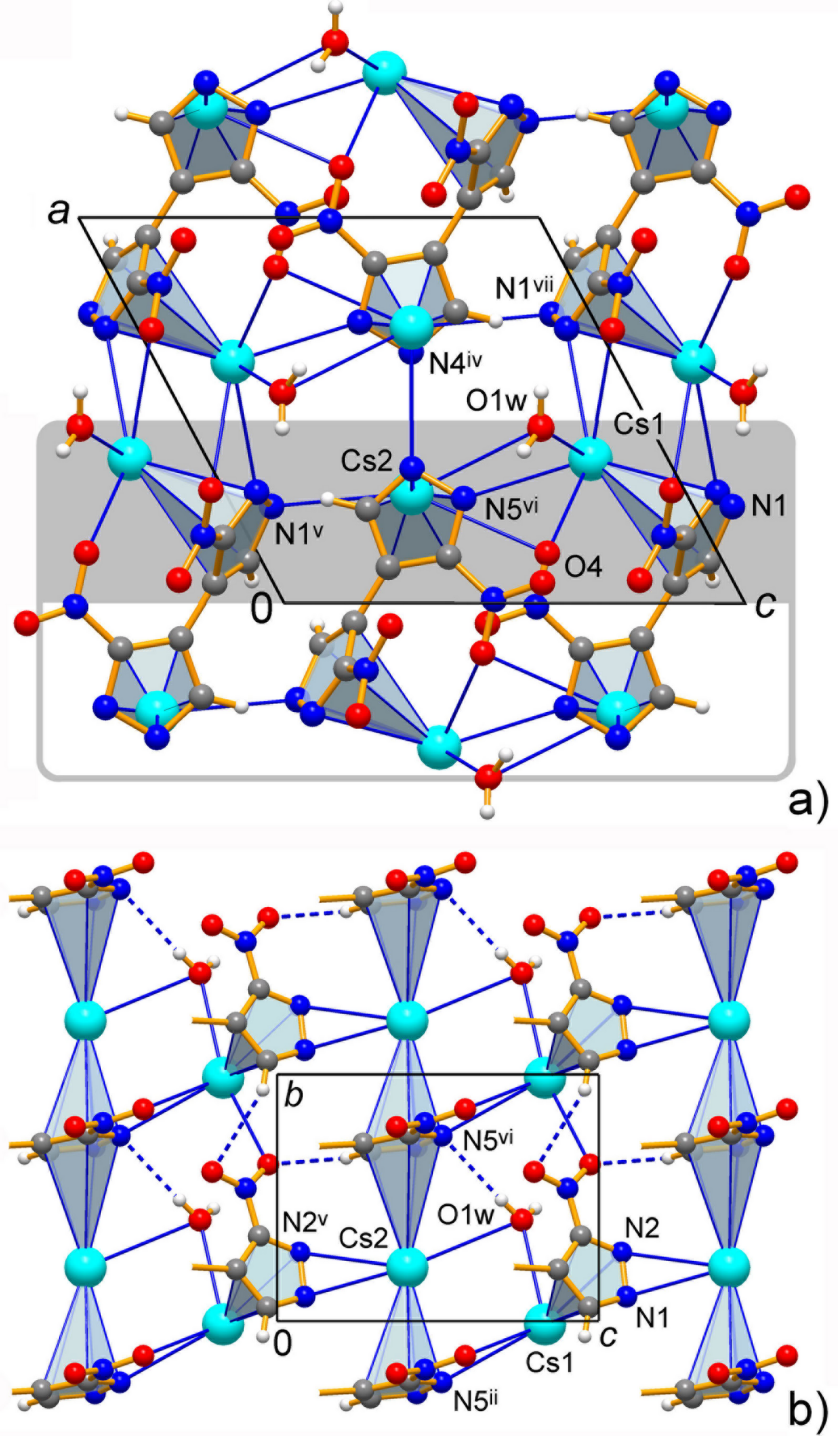
(*a*) Projection of the structure of **3** on the *ac* plane. The grey box indicates the nominal coordination layer, which is orthogonal to the drawing plane, and the grey-shaded area identifies the half-layer, which is further described (*b*) in its projection on the *bc* plane. Note the co-existence of the finite [Cs1] and chain-like [Cs2] π-coordination patterns. [Symmetry codes: (ii) −*x*, *y* − 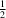, −*z* + 1; (v) *x*, *y*, *z* − 1; (vi) −*x*, *y* + 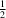, −*z* + 1; (vii) −*x* + 1, *y* + 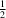, −*z* + 2.]

**Figure 7:**
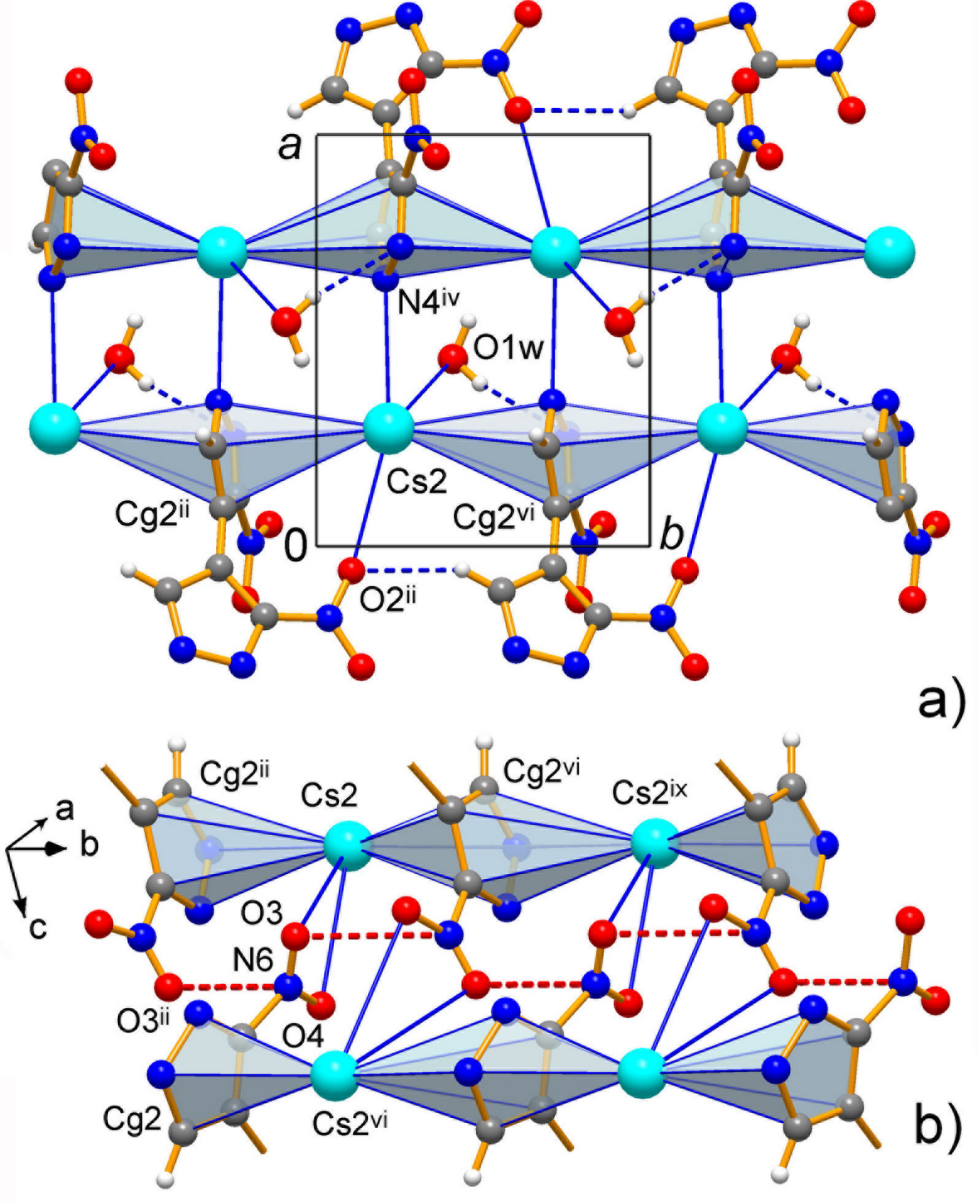
Two fragments of the structure of **3**, which depict one-dimensional Cs–π chains along the *b*-axis direction and their aggregation through reciprocal Cs—N bonds (*a*) and Cs—O bonds (*b*) leading to the generation of the infinite NO_2_/NO_2_ stack. [Symmetry codes: (ii) −*x*, *y* − 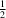, −*z* + 1; (iv) *x* + 1, *y*, *z*; (vi) −*x*, *y* + 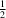, −*z* + 1; (ix) *x*, *y* + 1, *z*.]

The extended structure of **3** is completely different being dominated by cation–π bonding. We identify coordination layers lying parallel to the *bc* plane, in which the infinite Cs2–π(ring *B*)–Cs2–π(ring *B*) stacks along the *b*-axis direction are inter­linked through the Cs1–π(ring *A*) fragments with ring *A* coordinated to Cs2 in a κ^2^-fashion (Fig. 6 ▸), when two bond distances appear to be comparable in length [Cs2—N1^v^ = 3.251 (4) and Cs2—N2^v^ = 3.490 (4) Å; symmetry code: (v) *x*, *y*, *z* − 1.] However, attribution of the layers is only nominal, unlike **1** and **2**. There is no leading significance of *M*—N over *M*—O bonding and both kinds of conventional ionic inter­actions are equally important either for intra- or inter­layer connection. In this way, the above stacks afford two different motifs of mutual inter­actions (Fig. 7 ▸), by the reciprocal bonding of Cs ions and pyrazolate-N atoms between the layers [Cs—N4^iv^ = 3.132 (5) Å; symmetry code: (iv) *x* + 1, *y*, *z*)], while similar reciprocal coordination of pseudochelate nitro groups-O3/O4 unite the stacks within the layer [Cs2—O = 3.350 (5) and 3.502 (6) Å]. In addition, the water mol­ecules sit above and below the layers and they bridge the Cs1 and Cs2 ions with the comparable relatively long distances Cs—O = 3.215 (6) and 3.496 (7) Å and adopt an even more distal contact to the adjacent layer [Cs1—O1*w*^i^ = 3.754 (7) Å; symmetry code (i) −*x* + 1, *y* − 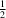, −*z* + 2]. They are also important as conventional hydrogen-bond donors to the most nucleophilic pyrazolate-N sites (Table 5 ▸), with the strongest bonds observed between the layers [O1*w*⋯N1^vii^ = 2.855 (7) Å, with a nearly linear angle at the H atom of 171°; symmetry code: (vii) −*x* + 1, *y* + 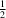, −*z* + 2]. The accessibility of pyrazolate-N acceptors for the hydrogen bonding is conditioned by the preferential Cs–π coordination over formation of Cs—N bonds. Therefore, the incorporation of water mol­ecules in **3**, in contrast to the formation of anhydrates **1** and **2**, may be regarded as a response to the needs for appropriate structural pairing of hard Lewis basic N atoms.

In addition to the main structure-defining Coulombic forces, ion–dipole bonding and conventional hydrogen bonding in **3**, the structures also reveal a variety of weak secondary inter­actions, which complement the coordination patterns. They include weak C—H⋯O hydrogen bonding with polarized pyrazole CH donors and nitro-O acceptors (Table 5 ▸) and different stacking inter­actions with the formation of either homo- (pyrazole/pyrazole, nitro/nitro) or heterofunctional pairs (pyrazole/nitro) (Table 6 ▸). The significance of weak hydrogen bonds is rather minor. All of them are found within the topological layers and they accompany the configurations imposed by coordination, with typical C⋯O separations in the range of 3.352 (4)–3.547 (9) Å (Desiraju & Steiner, 1999 ▸). The stacking of the rings is irrelevant for **3** since the axial positions at the pyrazole rings serve for the accommodation of Cs ions. Such inter­actions contribute to the suite of weak bonding inter­actions in the case of **1** and **2** between the coordination layers. In every case the π–π contacts are very distal, with the inter­centroid distances up to 3.967 (7) Å and relatively large slippage angles (Table 6 ▸). The *B* rings in **2** do not support overlap at all, but produce the relatively close stacks of pyrazole and NO_2_ groups with the nitro-N atoms residing exactly above the ring centroids at 3.402 (6) Å. Yet another kind of weak bond is the lone pair–π-hole inter­action (Bauzá *et al.*, 2017 ▸), which is equally relevant for each of the three structures, in the form of mutual NO_2_/NO_2_ stacking. Such inter­actions themselves could be superior in energetics to the common weak hydrogen bonds and they are one of the dominant factors for the crystal structures of polynitro species (Domasevitch *et al.*, 2020 ▸). However, in the present case these inter­actions are weak or very weak, as indicated by the corresponding N⋯O contacts at 3.142 (8)–3.354 (8) Å (Table 6 ▸), which are longer than the sum of van der Waals radii (3.07 Å).

## 4. Database survey

A search of the Cambridge Structural Database (CSD version 5.43, update of November 2022; Groom *et al.*, 2016 ▸) reveals no late alkali metal (K, Rb, Cs) mono­nitro­pyrazolates, while a series of the salts with different di­nitro­pyrazolate anions accounts for thirteen hits. Unless the nucleophilic N sites are blocked by strong N bonding, most of them display multiple pyrazolate-N coordinations, which is reminiscent of that observed for **1** and **2**. In particular, a rare mode μ_4_-κ^1^:κ^1^:κ^1^:κ^1^ was found for (μ-3,5-di­nitro­pyrazolid-4-olato)dicaesium (CSD refcode BEGXUK; Dong *et al.*, 2022 ▸), while its Rb analogue (BEGYAR; Dong *et al.*, 2022 ▸), (μ-3,4-di­nitro­pyrazolato)caesium (EDOTOK; Cao *et al.*, 2022 ▸) and (μ-3,5-di­nitro­pyrazolato)potassium (GIMPEA; Bölter *et al.*, 2018 ▸) provide tetra­dentate bridges of the type μ_3_-κ^2^:κ^1^:κ^1^. There are no unambiguous examples for the metal–π-nitro­pyrazolate coordination at all, which is not surprising for such electron-depleted systems. However, some cases revealed the metal ions situated nearly above the N atoms, at one of the axial sides of the ring. This may presumably be regarded as a very distal weak cation–π inter­action, which is accompanied with large slippage angles due to the significant shift of *M*^+^ toward more negatively polarized atoms. In this way, in GIMPEA the K^+^ to ring centroid distance is 3.41 Å, which is close to the parameters of **3** for the much larger Cs^+^ ion. A similar Rb⋯π contact of 3.40 Å is present in BEGYAR. Caesium 4-(pyrazol-4-yl)-3,5-di­nitro­pyrazolate monohydrate (FUFBIU; Gospodinov *et al.*, 2020 ▸) exhibits no Cs⋯π inter­actions to pyrazolate, but instead a very long such contact (3.67 Å) is found for the neutral pyrazol-4-yl ring. This feature disappears in the structure of the more electron-deficient tri­nitro analogue (FUFCIV; Gospodinov *et al.*, 2020 ▸).

## 5. Synthesis and crystallization

The ligand 3,3′-di­nitro-4,4′-bi­pyrazole was prepared in 79% yield by nitration of 4,4′-bi­pyrazole in phospho­ric acid (Domasevitch *et al.*, 2019 ▸). This synthesis is not routine since the common nitration of the substrate in mixed acids proceeds through attack on a deactivated pyrazolium cation (Austin *et al.*, 1965 ▸), but the mononitrated ring readily undergoes second substitution as more reactive free base. This results in the apparent paradox of dinitration at the same ring with the production of isomeric 3,5-di­nitro-4,4′-bi­pyrazole. Therefore the utilization of less acidic H_3_PO_4_ is a key pre-requisite for the success of preparation.

To prepare the alkali metal salts, 34 mg (0.15 mmol) of 3,3′-di­nitro-4,4′-bi­pyrazole were dissolved in 3 ml of 20% aqueous solution of alkali metal (K, Rb or Cs) hydroxide under stirring and heating to 333–343 K. The resulting clear dark-red solution was left overnight, after which the crystalline deposit of orange–red bipyrazolate salt (yields 70–80%) was filtered and washed with 1–2 ml of 2-propanol.

Analysis (%) calculated for (**1**), C_6_H_2_K_2_N_6_O_4_: C 23.99, H 0.67, N 27.99; found: C 23.67, H 0.81, N 27.70. Analysis (%) calculated for (**2**), C_6_H_2_N_6_O_4_Rb_2_: C 18.33, H 0.51, N 21.39; found: C 18.27, H 0.68, N 21.08. Analysis (%) calculated for (**3**), C_6_H_4_Cs_2_N_6_O_5_: C 14.24, H 0.80, N 16.61; found: C 14.02, H 0.88, N 16.38.

## 6. Refinement

Crystal data, data collection and structure refinement details are summarized in Table 7 ▸. Structures **1** and **3** were refined as inversion twins with partial contribution factors 0.587/0.413 and 0.563/0.437, respectively. The water H atoms in **3** were located and then restrained with O—H = 0.85 Å and *U*_iso_ = 1.5*U*_eq_ (carrier O-atom), whereas all C-bound hydrogen atoms were constrained geometrically and refined as riding with *U*_iso_(H) = 1.2*U*_eq_(carrier C-atom).

## Supplementary Material

Crystal structure: contains datablock(s) global, 1, 2, 3. DOI: 10.1107/S2056989026005037/hb8215sup1.cif

Structure factors: contains datablock(s) 1. DOI: 10.1107/S2056989026005037/hb82151sup2.hkl

Structure factors: contains datablock(s) 2. DOI: 10.1107/S2056989026005037/hb82152sup3.hkl

Structure factors: contains datablock(s) 3. DOI: 10.1107/S2056989026005037/hb82153sup4.hkl

CCDC references: 2553747, 2553746, 2553745

Additional supporting information:  crystallographic information; 3D view; checkCIF report

## Figures and Tables

**Figure 2:**
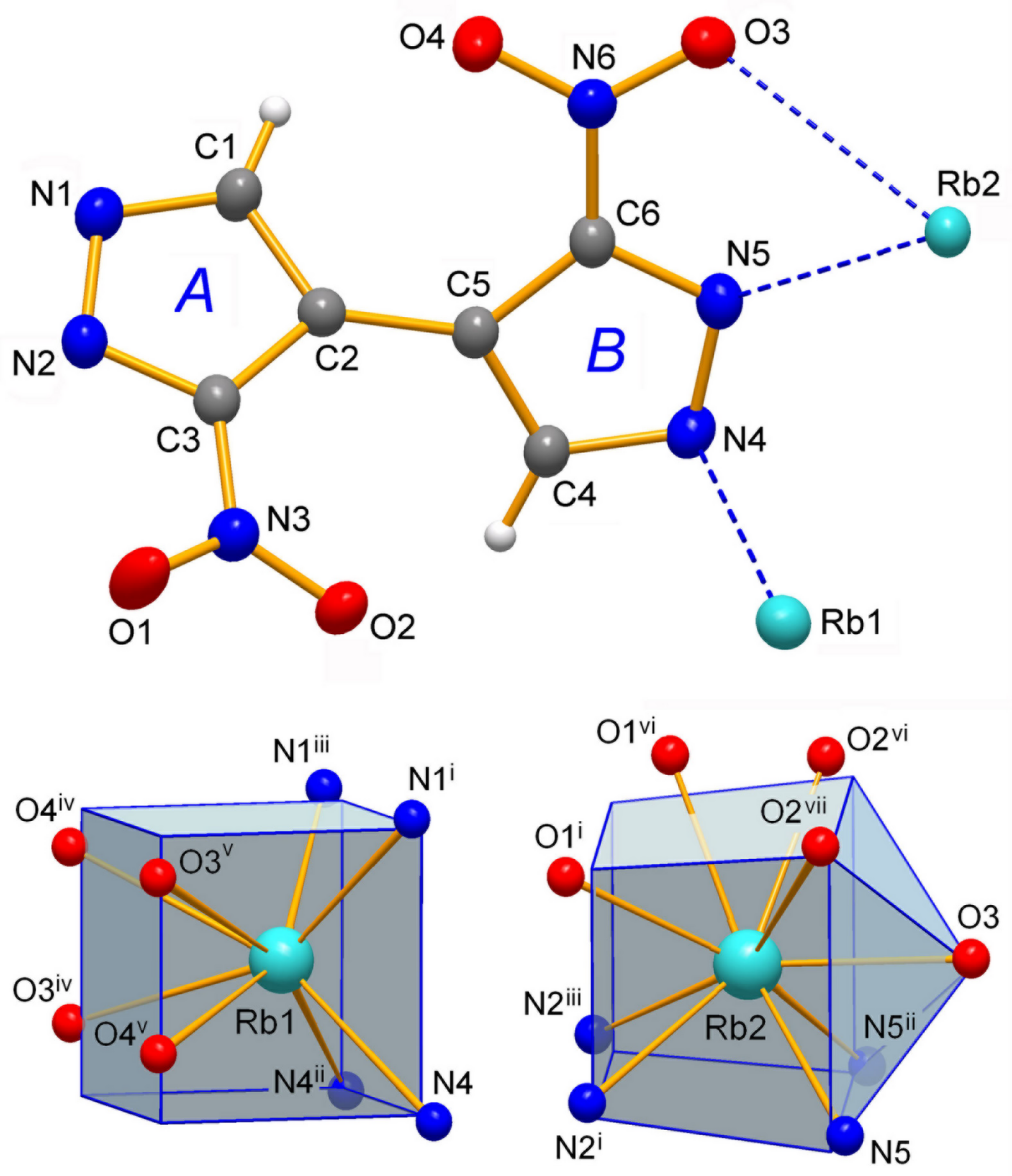
The mol­ecular structure of **2** with displacement ellipsoids at the 50% probability level. The coordination environments of two metal ions are drawn against the best fitted idealized polyhedra in the form of cube (Rb1) and spherical-relaxed capped cube (Rb2). For symmetry codes, see Table 2 ▸.

**Table 1:** Selected bond lengths (Å) for **1**[Chem scheme1]

K1—O2^i^	2.719 (2)	K2—O4^vi^	2.853 (2)
K1—N1^ii^	2.783 (3)	K2—N2	2.859 (3)
K1—O3^iii^	2.795 (2)	K2—N4^vii^	2.901 (3)
K1—N5^iii^	2.828 (3)	K2—N2^viii^	2.917 (3)
K1—N5^iv^	2.850 (3)	K2—O1^viii^	2.945 (2)
K1—N1	2.871 (3)	K2—N4^iv^	2.956 (3)
K1—O1^i^	3.091 (3)	K2—O4^ix^	2.973 (3)
K1—O2^v^	3.418 (3)	K2—O3^vi^	3.153 (2)

Symmetry codes: (i) 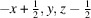; (ii) 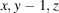; (iii) 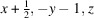; (iv) 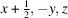; (v) 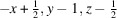; (vi) 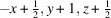; (vii) 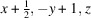; (viii) 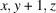; (ix) 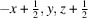.

**Table 2:** Selected bond lengths (Å) for **2**[Chem scheme1]

Rb1—N1^i^	2.930 (4)	Rb2—O1^vi^	2.993 (4)
Rb1—N4^ii^	2.980 (4)	Rb2—O3	3.004 (4)
Rb1—N1^iii^	2.987 (4)	Rb2—N5	3.016 (4)
Rb1—N4	3.088 (4)	Rb2—N5^ii^	3.017 (4)
Rb1—O3^iv^	3.103 (4)	Rb2—N2^iii^	3.101 (4)
Rb1—O3^v^	3.113 (4)	Rb2—O2^vi^	3.150 (4)
Rb1—O4^iv^	3.175 (4)	Rb2—N2^i^	3.194 (4)
Rb1—O4^v^	3.370 (5)	Rb2—O2^vii^	3.214 (4)
Rb2—O1^i^	2.893 (4)		

Symmetry codes: (i) 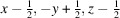; (ii) 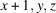; (iii) 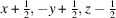; (iv) 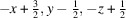; (v) 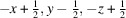; (vi) 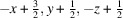; (vii) 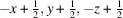.

**Table 3:** Selected bond lengths (Å) for **3**[Chem scheme1]

Cs1—O1^i^	3.064 (5)	Cs2—O2^ii^	3.107 (5)
Cs1—O3^ii^	3.101 (6)	Cs2—N4^iv^	3.132 (5)
Cs1—O4	3.123 (5)	Cs2—N1^v^	3.251 (6)
Cs1—O1^iii^	3.130 (6)	Cs2—O4	3.350 (5)
Cs1—O1*W*	3.215 (6)	Cs2—O1*W*	3.496 (7)
Cs1—N2^i^	3.273 (5)	Cs2—O3	3.502 (6)
Cs1—N5^ii^	3.392 (6)	Cs2—*Cg*2^ii^	3.474 (3)
Cs1—*Cg*1	3.389 (3)	Cs2—*Cg*2^vi^	3.587 (3)
Cs1—O1*W*^i^	3.754 (7)		

Symmetry codes: (i) 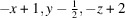; (ii) 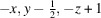; (iii) 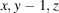; (iv) 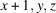; (v) 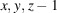; (vi) 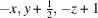.

**Table 4:** Geometry of the Cs–π-pyrazolate coordination in **3** (Å, °) Cs⋯Plane is the distance from the metal ion to the mean plane of the ring and sa is the slippage angle, *i.e.* the angle of the Cs⋯*Cg* axis to the plane normal.

Ion	Group	Cs—N	Cs—C	Mean Cs—C,*N*	Cs⋯*Cg*	Cs⋯Plane	sa
Cs1	(N1/N2/C1–3)	3.433 (4), 3.562 (4)	3.514 (5)–3.703 (4)	3.581 (5)	3.389 (3)	3.360 (2)	7.5 (3)
Cs2	(N4/N5/C4–6)^ii^	3.442 (5), 3.589 (6)	3.588 (5)–3.868 (5)	3.661 (6)	3.474 (3)	3.406 (3)	11.4 (4)
	(N4/N5/C4–6)^vi^	3.451 (6), 3.896 (5)	3.448 (6)–4.111 (5)	3.762 (6)	3.587 (3)	3.369 (5)	20.1 (4)

Symmetry codes: (ii) −*x*, *y* − 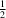, −*z* + 1; (vi) −*x*, *y* + 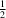, −*z* + 1.

**Table 5:** Geometry of hydrogen bonding (Å, °) for **1**–**3**

Compound	*D*—H⋯*A*	*D*—H	H⋯*A*	*D*⋯*A*	*D*—H⋯*A*
**1**	C4—H4⋯O3^*x*^	0.94	2.62	3.352 (4)	135
**2**	C4—H4⋯O2^viii^	0.95	2.68	3.506 (6)	146
**3**	O1w—H1*w*⋯N1^vii^	0.85	2.01	2.855 (7)	171
	O1w—H2*w*⋯N5^vi^	0.85	2.50	3.334 (9)	168
	C1—H1⋯O2^iii^	0.94	2.83	3.508 (10)	130
	C4—H4⋯O1^viii^	0.94	2.62	3.547 (9)	168

Symmetry codes for **1**: (*x*) −*x*, −*y*, *z* + 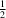; for **2**: (viii) *x* − 1, *y*, *z*; for **3**: (iii) *x*, *y* − 1, *z*; (vi) −*x*, *y* + 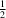, −*z* + 1; (vii) −*x* + 1, *y* + 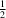, −*z* + 2; (viii) −*x*, *y* − 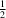, −*z* + 2.

**Table 6:** Geometry of stacking inter­actions (Å, °) for **1**–**3** *Cg*1⋯*Cg*2 is the distance between the centroids of Group 1 and Group 2; *Cg*1⋯·Plane is the distance from the Group 1 centroid to the mean plane of Group 2 or the distance of an O-donor to the mean plane of a nitro group; sa is the slippage angle *i.e.* the angle of the *Cg*1⋯*Cg*2 axis to the plane of Group 2 or the angle of the O⋯N axis to the plane of the nitro group for the NO_2_/NO_2_ patterns.

Compound	Type	Group 1	Group 2	Shortest contact	*Cg*1⋯*Cg*2	*Cg*1⋯Plane	sa
**1**	Pyrazole/Pyrazole	(N1/N2/C1–3)	(N1/N2/C1–3)^ii^	3.492 (3)	3.768 (3)	3.479 (4)	22.6 (4)
	Pyrazole/Pyrazole	(N4/N5/C4–6)	(N4/N5/C4–6)^ii^	3.456 (3)	3.768 (3)	3.470 (4)	22.9 (4)
	NO_2_/NO_2_	(C3/N3/O1/O2)	(C3/N3/O1/O2)^ii^	3.238 (3)	–	3.104 (3)	16.5 (5)
	NO_2_/NO_2_	(C6/N6/O3/O4)	(C6/N6/O3/O4)^ii^	3.298 (3)	–	3.176 (4)	15.6 (6)
**2**	Pyrazole/Pyrazole	(N1/N2/C1–3)	(N1/N2/C1–3)^ii^	3.707 (7)	3.967 (6)	3.689 (7)	21.6 (7)
	Pyrazole/NO_2_	(N4/N5/C4–6)	(C6/N6/O3/O4)^viii^	3.375 (6)	3.402 (6)	3.374 (7)	7.4 (8)
	NO_2_/NO_2_	(C3/N3/O1/O2)	(C3/N3/O1/O2)^ii^	3.354 (8)	–	3.150 (8)	20.1 (8)
**3**	NO_2_/NO_2_	(C6/N6/O3/O4)	(C6/N6/O3/O4)^vi^	3.142 (8)	–	2.871 (9)	24.0 (8)

Symmetry codes for **1**: (ii) *x*, *y* − 1, *z*; for **2**:(ii) *x* + 1, *y*, *z*; (viii) *x* − 1, *y*, *z*; for **3**: (vi) −*x*, *y* + 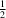, −*z* + 1.

**Table 7:** Experimental details

	**1**	**2**	**3**
Crystal data			
Chemical formula	[K_2_(C_6_H_2_N_6_O_4_)]	[Rb_2_(C_6_H_2_N_6_O_4_)]	[Cs_2_(C_6_H_2_N_6_O_4_)]·H_2_O
*M*_r_	300.34	393.08	505.97
Crystal system, space group	Orthorhombic, *Pca*2_1_	Monoclinic, *P*2_1_/*n*	Monoclinic, *P*2_1_
Temperature (K)	213	173	213
*a*, *b*, *c* (Å)	18.7276 (19), 3.7683 (3), 13.9808 (13)	3.9669 (2), 14.2020 (4), 18.6685 (6)	9.7388 (7), 6.9551 (3), 10.2950 (7)
α, β, γ (°)	90, 90, 90	90, 95.776 (3), 90	90, 118.152 (8), 90
*V* (Å^3^)	986.64 (16)	1046.40 (7)	614.83 (8)
*Z*	4	4	2
Radiation type	Mo *K*α	Cu *K*α	Mo *K*α
μ (mm^−1^)	0.98	12.38	5.96
Crystal size (mm)	0.22 × 0.21 × 0.18	0.05 × 0.03 × 0.03	0.20 × 0.17 × 0.14

Data collection			
Diffractometer	Stoe Image plate diffraction system-2T	Stoe Stadivari	Stoe Image plate diffraction system-2T
Absorption correction	Numerical [*X-RED* (Stoe & Cie, 2001 ▸) and *X-SHAPE* (Stoe & Cie, 1999 ▸)]	Multi-scan (Stoe *LANA*; Koziskova *et al.*, 2016 ▸)	Numerical [*X-RED* (Stoe & Cie, 2001 ▸) and *X-SHAPE* (Stoe & Cie, 1999 ▸)]
*T*_min_, *T*_max_	0.854, 0.871	0.469, 0.661	0.218, 0.245
No. of measured, independent and observed [*I* > 2σ(*I*)] reflections	6480, 2285, 1849	10655, 2234, 2156	4971, 2554, 2376
*R*_int_	0.022	0.030	0.020
(sin θ/λ)_max_ (Å^−1^)	0.660	0.638	0.641

Refinement			
*R*[*F*^2^ > 2σ(*F*^2^)], *wR*(*F*^2^), *S*	0.021, 0.048, 0.91	0.048, 0.148, 1.14	0.020, 0.045, 0.99
No. of reflections	2285	2234	2554
No. of parameters	164	164	173
No. of restraints	1	0	1
H-atom treatment	H-atom parameters constrained	H-atom parameters constrained	H-atom parameters constrained
Δρ_max_, Δρ_min_ (e Å^−3^)	0.22, −0.19	0.86, −0.73	0.93, −1.17
Absolute structure	Refined as an inversion twin	–	Refined as an inversion twin
Absolute structure parameter	0.41 (4)	–	0.44 (3)

Computer programs: *IPDS Software* (Stoe & Cie, 2000 ▸), *X-AREA 1.76* (Stoe & Cie, 2016 ▸), *SHELXS97* (Sheldrick, 2008 ▸), *SHELXL2019/3* (Sheldrick, 2015 ▸), *DIAMOND 3.0* (Brandenburg, 1999 ▸) and *WinGX 1.70.01* (Farrugia, 2012 ▸).

## supplementary crystallographic information

### Poly[[µ-3,3'-dinitro-4,4'-bipyrazole-1,1'-diido]dipotassium] (1) Crystal data

**Table:**

[K_2_(C_6_H_2_N_6_O_4_)]	*D*_x_ = 2.022 Mg m^−^^3^
*M**_r_* = 300.34	Mo *K*α radiation, λ = 0.71073 Å
Orthorhombic, *P**c**a*2_1_	Cell parameters from 6480 reflections
*a* = 18.7276 (19) Å	θ = 2.6–28.0°
*b* = 3.7683 (3) Å	µ = 0.98 mm^−^^1^
*c* = 13.9808 (13) Å	*T* = 213 K
*V* = 986.64 (16) Å^3^	Prism, red
*Z* = 4	0.22 × 0.21 × 0.18 mm
*F*(000) = 600	

### Poly[[µ-3,3'-dinitro-4,4'-bipyrazole-1,1'-diido]dipotassium] (1) Data collection

**Table:**

Stoe Image plate diffraction system-2T diffractometer	1849 reflections with *I* > 2σ(*I*)
Radiation source: fine-focus sealed tube	*R*_int_ = 0.022
φ oscillation scans	θ_max_ = 28.0°, θ_min_ = 2.6°
Absorption correction: numerical [X-RED (Stoe & Cie, 2001) and X-SHAPE (Stoe & Cie, 1999)]	*h* = −24→24
*T*_min_ = 0.854, *T*_max_ = 0.871	*k* = −4→4
6480 measured reflections	*l* = −18→18
2285 independent reflections	

### Poly[[µ-3,3'-dinitro-4,4'-bipyrazole-1,1'-diido]dipotassium] (1) Refinement

**Table:**

Refinement on *F*^2^	Secondary atom site location: difference Fourier map
Least-squares matrix: full	Hydrogen site location: inferred from neighbouring sites
*R*[*F*^2^ > 2σ(*F*^2^)] = 0.021	H-atom parameters constrained
*wR*(*F*^2^) = 0.048	*w* = 1/[σ^2^(*F*_o_^2^) + (0.0272*P*)^2^] where *P* = (*F*_o_^2^ + 2*F*_c_^2^)/3
*S* = 0.91	(Δ/σ)_max_ < 0.001
2285 reflections	Δρ_max_ = 0.22 e Å^−^^3^
164 parameters	Δρ_min_ = −0.19 e Å^−^^3^
1 restraint	Absolute structure: Refined as an inversion twin
Primary atom site location: structure-invariant direct methods	Absolute structure parameter: 0.41 (4)

### Poly[[µ-3,3'-dinitro-4,4'-bipyrazole-1,1'-diido]dipotassium] (1) Special details

**Table:**

Geometry. All esds (except the esd in the dihedral angle between two l.s. planes) are estimated using the full covariance matrix. The cell esds are taken into account individually in the estimation of esds in distances, angles and torsion angles; correlations between esds in cell parameters are only used when they are defined by crystal symmetry. An approximate (isotropic) treatment of cell esds is used for estimating esds involving l.s. planes.
Refinement. Refined as a 2-component inversion twin.

### Poly[[µ-3,3'-dinitro-4,4'-bipyrazole-1,1'-diido]dipotassium] (1) Fractional atomic coordinates and isotropic or equivalent isotropic displacement parameters (Å^2^)

**Table:**

	*x*	*y*	*z*	*U*_iso_*/*U*_eq_	
K1	0.37544 (4)	−0.49087 (18)	0.05811 (4)	0.02022 (13)	
K2	0.37880 (3)	0.37981 (18)	0.32366 (3)	0.02035 (14)	
O1	0.23811 (12)	−0.4115 (7)	0.39913 (17)	0.0331 (6)	
O2	0.13683 (12)	−0.1418 (9)	0.38871 (16)	0.0418 (7)	
O3	0.00886 (11)	−0.3670 (7)	−0.02285 (16)	0.0301 (5)	
O4	0.11495 (11)	−0.1447 (7)	−0.01775 (15)	0.0323 (6)	
N1	0.28453 (13)	0.0267 (7)	0.1470 (2)	0.0210 (6)	
N2	0.28173 (12)	−0.1085 (8)	0.23656 (18)	0.0186 (5)	
N3	0.19516 (12)	−0.2269 (8)	0.35423 (17)	0.0220 (6)	
N4	−0.02670 (12)	0.1315 (8)	0.22521 (17)	0.0194 (5)	
N5	−0.02718 (13)	−0.0134 (7)	0.1359 (2)	0.0193 (6)	
N6	0.05558 (13)	−0.1962 (7)	0.01939 (16)	0.0203 (6)	
C1	0.21717 (15)	0.1044 (9)	0.1183 (2)	0.0194 (6)	
H1	0.205558	0.199449	0.058094	0.023*	
C2	0.16761 (15)	0.0263 (9)	0.1887 (2)	0.0158 (9)	
C3	0.21248 (14)	−0.1040 (9)	0.2612 (2)	0.0162 (6)	
C4	0.04169 (14)	0.1663 (9)	0.2536 (2)	0.0186 (6)	
H4	0.055404	0.256931	0.313491	0.022*	
C5	0.08971 (15)	0.0525 (10)	0.18402 (19)	0.0146 (7)	
C6	0.04176 (14)	−0.0536 (8)	0.1116 (2)	0.0153 (6)	

### Poly[[µ-3,3'-dinitro-4,4'-bipyrazole-1,1'-diido]dipotassium] (1) Atomic displacement parameters (Å^2^)

**Table:**

	*U*^11^	*U*^22^	*U*^33^	*U*^12^	*U*^13^	*U*^23^
K1	0.0164 (2)	0.0229 (3)	0.0214 (3)	0.0004 (2)	0.0000 (3)	0.0051 (2)
K2	0.0201 (3)	0.0204 (4)	0.0206 (3)	−0.0003 (3)	−0.0016 (3)	−0.0004 (2)
O1	0.0296 (12)	0.0443 (17)	0.0253 (12)	0.0093 (11)	−0.0059 (10)	0.0116 (10)
O2	0.0216 (11)	0.076 (2)	0.0273 (12)	0.0113 (12)	0.0091 (9)	0.0202 (12)
O3	0.0267 (11)	0.0450 (16)	0.0186 (10)	−0.0112 (11)	−0.0024 (9)	−0.0077 (10)
O4	0.0203 (11)	0.0539 (17)	0.0227 (10)	−0.0065 (11)	0.0068 (9)	−0.0112 (10)
N1	0.0146 (12)	0.0240 (19)	0.0244 (15)	−0.0021 (10)	0.0025 (10)	0.0002 (10)
N2	0.0114 (11)	0.0229 (16)	0.0216 (13)	−0.0013 (10)	0.0007 (9)	−0.0007 (11)
N3	0.0181 (12)	0.0302 (17)	0.0178 (11)	−0.0020 (10)	−0.0024 (9)	0.0016 (10)
N4	0.0143 (11)	0.0253 (17)	0.0187 (12)	0.0020 (10)	0.0027 (9)	−0.0019 (12)
N5	0.0154 (11)	0.0219 (18)	0.0207 (14)	0.0001 (9)	−0.0016 (9)	−0.0005 (10)
N6	0.0184 (12)	0.0258 (17)	0.0167 (11)	0.0014 (10)	−0.0012 (9)	0.0016 (10)
C1	0.0173 (14)	0.0224 (18)	0.0186 (14)	−0.0014 (12)	0.0020 (10)	0.0016 (13)
C2	0.0133 (12)	0.015 (2)	0.0191 (18)	−0.0002 (11)	−0.0021 (11)	−0.0022 (10)
C3	0.0139 (13)	0.0183 (17)	0.0163 (15)	−0.0010 (11)	−0.0002 (10)	0.0002 (12)
C4	0.0151 (12)	0.022 (2)	0.0189 (14)	0.0023 (11)	0.0004 (10)	−0.0021 (11)
C5	0.0149 (12)	0.016 (2)	0.0127 (16)	−0.0005 (13)	0.0020 (10)	0.0025 (10)
C6	0.0123 (13)	0.0170 (18)	0.0167 (15)	0.0007 (10)	−0.0004 (10)	0.0009 (11)

### Poly[[µ-3,3'-dinitro-4,4'-bipyrazole-1,1'-diido]dipotassium] (1) Geometric parameters (Å, º)

**Table:**

K1—O2^i^	2.719 (2)	O3—N6	1.236 (3)
K1—N1^ii^	2.783 (3)	O4—N6	1.242 (3)
K1—O3^iii^	2.795 (2)	N1—N2	1.353 (4)
K1—N5^iii^	2.828 (3)	N1—C1	1.356 (4)
K1—N5^iv^	2.850 (3)	N2—C3	1.342 (3)
K1—N1	2.871 (3)	N3—C3	1.418 (4)
K1—O1^i^	3.091 (3)	N4—C4	1.347 (4)
K1—O2^v^	3.418 (3)	N4—N5	1.362 (4)
K2—O4^vi^	2.853 (2)	N5—C6	1.344 (4)
K2—N2	2.859 (3)	N6—C6	1.420 (4)
K2—N4^vii^	2.901 (3)	C1—C2	1.385 (4)
K2—N2^viii^	2.917 (3)	C1—H1	0.9400
K2—O1^viii^	2.945 (2)	C2—C3	1.406 (4)
K2—N4^iv^	2.956 (3)	C2—C5	1.464 (3)
K2—O4^ix^	2.973 (3)	C4—C5	1.393 (4)
K2—O3^vi^	3.153 (2)	C4—H4	0.9400
O1—N3	1.235 (3)	C5—C6	1.411 (4)
O2—N3	1.236 (3)		
			
O2^i^—K1—N1^ii^	130.77 (8)	N6—O4—K2^i^	116.80 (18)
O2^i^—K1—O3^iii^	79.37 (7)	K2^v^—O4—K2^i^	80.58 (6)
N1^ii^—K1—O3^iii^	127.04 (8)	N2—N1—C1	108.6 (2)
O2^i^—K1—N5^iii^	135.17 (8)	N2—N1—K1^viii^	133.1 (2)
N1^ii^—K1—N5^iii^	77.95 (8)	C1—N1—K1^viii^	107.2 (2)
O3^iii^—K1—N5^iii^	56.82 (7)	N2—N1—K1	99.67 (19)
O2^i^—K1—N5^iv^	93.68 (8)	C1—N1—K1	124.8 (2)
N1^ii^—K1—N5^iv^	131.04 (8)	K1^viii^—N1—K1	83.56 (7)
O3^iii^—K1—N5^iv^	73.16 (8)	C3—N2—N1	105.7 (2)
N5^iii^—K1—N5^iv^	83.16 (7)	C3—N2—K2	119.8 (2)
O2^i^—K1—N1	89.90 (9)	N1—N2—K2	97.32 (19)
N1^ii^—K1—N1	83.56 (7)	C3—N2—K2^ii^	120.2 (2)
O3^iii^—K1—N1	146.67 (8)	N1—N2—K2^ii^	127.67 (18)
N5^iii^—K1—N1	131.64 (7)	K2—N2—K2^ii^	81.45 (7)
N5^iv^—K1—N1	76.19 (7)	O1—N3—O2	121.6 (3)
O2^i^—K1—O1^i^	42.99 (6)	O1—N3—C3	120.1 (2)
N1^ii^—K1—O1^i^	87.90 (7)	O2—N3—C3	118.4 (3)
O3^iii^—K1—O1^i^	110.02 (8)	O1—N3—K1^ix^	69.62 (16)
N5^iii^—K1—O1^i^	141.65 (8)	O2—N3—K1^ix^	52.17 (15)
N5^iv^—K1—O1^i^	130.57 (7)	C3—N3—K1^ix^	169.57 (18)
N1—K1—O1^i^	80.64 (8)	C4—N4—N5	108.4 (2)
O2^i^—K1—O2^v^	74.80 (6)	C4—N4—K2^xi^	112.22 (19)
N1^ii^—K1—O2^v^	78.43 (7)	N5—N4—K2^xi^	133.24 (19)
O3^iii^—K1—O2^v^	69.01 (7)	C4—N4—K2^xii^	119.7 (2)
N5^iii^—K1—O2^v^	80.56 (7)	N5—N4—K2^xii^	99.29 (18)
N5^iv^—K1—O2^v^	141.84 (7)	K2^xi^—N4—K2^xii^	80.08 (6)
N1—K1—O2^v^	138.37 (7)	C4—N4—K1^xii^	134.8 (2)
O1^i^—K1—O2^v^	61.63 (7)	N5—N4—K1^xii^	60.45 (15)
O4^vi^—K2—N2	139.58 (7)	K2^xi^—N4—K1^xii^	74.55 (6)
O4^vi^—K2—N4^vii^	86.85 (7)	K2^xii^—N4—K1^xii^	105.52 (7)
N2—K2—N4^vii^	126.46 (8)	C6—N5—N4	105.7 (2)
O4^vi^—K2—N2^viii^	86.26 (7)	C6—N5—K1^x^	116.6 (2)
N2—K2—N2^viii^	81.45 (7)	N4—N5—K1^x^	128.42 (19)
N4^vii^—K2—N2^viii^	76.26 (7)	C6—N5—K1^xii^	126.4 (2)
O4^vi^—K2—O1^viii^	65.83 (7)	N4—N5—K1^xii^	94.98 (18)
N2—K2—O1^viii^	75.85 (7)	K1^x^—N5—K1^xii^	83.16 (7)
N4^vii^—K2—O1^viii^	123.10 (8)	C6—N5—K2^xii^	129.7 (2)
N2^viii^—K2—O1^viii^	54.23 (7)	N4—N5—K2^xii^	57.76 (15)
O4^vi^—K2—N4^iv^	138.30 (7)	K1^x^—N5—K2^xii^	72.49 (6)
N2—K2—N4^iv^	76.28 (7)	K1^xii^—N5—K2^xii^	103.34 (8)
N4^vii^—K2—N4^iv^	80.08 (6)	O3—N6—O4	121.0 (2)
N2^viii^—K2—N4^iv^	127.57 (7)	O3—N6—C6	120.1 (2)
O1^viii^—K2—N4^iv^	151.21 (8)	O4—N6—C6	118.9 (2)
O4^vi^—K2—O4^ix^	80.58 (6)	O3—N6—K2^v^	68.08 (15)
N2—K2—O4^ix^	85.11 (7)	O4—N6—K2^v^	54.09 (14)
N4^vii^—K2—O4^ix^	138.75 (7)	C6—N6—K2^v^	167.03 (18)
N2^viii^—K2—O4^ix^	140.86 (7)	N1—C1—C2	111.6 (3)
O1^viii^—K2—O4^ix^	86.88 (6)	N1—C1—H1	124.2
N4^iv^—K2—O4^ix^	83.71 (7)	C2—C1—H1	124.2
O4^vi^—K2—O3^vi^	41.73 (6)	K1^viii^—C1—H1	79.1
N2—K2—O3^vi^	155.36 (7)	C1—C2—C3	100.7 (2)
N4^vii^—K2—O3^vi^	73.96 (7)	C1—C2—C5	128.5 (2)
N2^viii^—K2—O3^vi^	120.00 (7)	C3—C2—C5	130.7 (3)
O1^viii^—K2—O3^vi^	105.80 (7)	N2—C3—C2	113.4 (3)
N4^iv^—K2—O3^vi^	96.58 (6)	N2—C3—N3	116.9 (3)
O4^ix^—K2—O3^vi^	70.57 (6)	C2—C3—N3	129.7 (2)
N3—O1—K2^ii^	123.44 (18)	N4—C4—C5	112.2 (3)
N3—O1—K1^ix^	88.39 (16)	N4—C4—H4	123.9
K2^ii^—O1—K1^ix^	147.93 (8)	C5—C4—H4	123.9
N3—O2—K1^ix^	106.79 (19)	C4—C5—C6	100.2 (2)
N3—O2—K1^vi^	121.0 (2)	C4—C5—C2	129.3 (2)
K1^ix^—O2—K1^vi^	74.80 (6)	C6—C5—C2	130.3 (3)
N6—O3—K1^x^	122.62 (17)	N5—C6—C5	113.4 (3)
N6—O3—K2^v^	90.59 (16)	N5—C6—N6	116.6 (3)
K1^x^—O3—K2^v^	144.50 (9)	C5—C6—N6	130.0 (3)
N6—O4—K2^v^	105.26 (17)		
			
C1—N1—N2—C3	−1.1 (4)	K1^viii^—C1—C2—C3	−49.2 (5)
K1^viii^—N1—N2—C3	136.8 (2)	N1—C1—C2—C5	−176.2 (4)
K1—N1—N2—C3	−133.0 (2)	K1^viii^—C1—C2—C5	134.4 (4)
C1—N1—N2—K2	−124.9 (2)	N1—N2—C3—C2	1.2 (4)
K1^viii^—N1—N2—K2	13.0 (3)	K2—N2—C3—C2	109.5 (3)
K1—N1—N2—K2	103.18 (11)	K2^ii^—N2—C3—C2	−152.7 (2)
C1—N1—N2—K2^ii^	150.3 (2)	N1—N2—C3—N3	−178.7 (3)
K1^viii^—N1—N2—K2^ii^	−71.8 (4)	K2—N2—C3—N3	−70.4 (3)
K1—N1—N2—K2^ii^	18.4 (3)	K2^ii^—N2—C3—N3	27.4 (4)
K2^ii^—O1—N3—O2	−179.3 (2)	C1—C2—C3—N2	−0.8 (4)
K1^ix^—O1—N3—O2	4.9 (3)	C5—C2—C3—N2	175.4 (4)
K2^ii^—O1—N3—C3	0.1 (4)	C1—C2—C3—N3	179.0 (4)
K1^ix^—O1—N3—C3	−175.7 (3)	C5—C2—C3—N3	−4.8 (6)
K2^ii^—O1—N3—K1^ix^	175.8 (2)	O1—N3—C3—N2	−18.3 (5)
K1^ix^—O2—N3—O1	−5.9 (4)	O2—N3—C3—N2	161.1 (3)
K1^vi^—O2—N3—O1	76.2 (4)	K1^ix^—N3—C3—N2	−175.5 (10)
K1^ix^—O2—N3—C3	174.8 (2)	O1—N3—C3—C2	161.9 (3)
K1^vi^—O2—N3—C3	−103.1 (3)	O2—N3—C3—C2	−18.7 (5)
K1^vi^—O2—N3—K1^ix^	82.07 (14)	K1^ix^—N3—C3—C2	4.6 (15)
C4—N4—N5—C6	−1.6 (4)	N5—N4—C4—C5	1.1 (4)
K2^xi^—N4—N5—C6	147.6 (2)	K2^xi^—N4—C4—C5	−155.2 (2)
K2^xii^—N4—N5—C6	−127.3 (2)	K2^xii^—N4—C4—C5	113.8 (3)
K1^xii^—N4—N5—C6	130.0 (2)	K1^xii^—N4—C4—C5	−65.2 (4)
C4—N4—N5—K1^x^	143.1 (2)	N4—C4—C5—C6	−0.1 (4)
K2^xi^—N4—N5—K1^x^	−67.6 (3)	N4—C4—C5—C2	−175.9 (4)
K2^xii^—N4—N5—K1^x^	17.4 (2)	C1—C2—C5—C4	−140.9 (4)
K1^xii^—N4—N5—K1^x^	−85.21 (19)	C3—C2—C5—C4	43.9 (6)
C4—N4—N5—K1^xii^	−131.7 (2)	C1—C2—C5—C6	44.5 (6)
K2^xi^—N4—N5—K1^xii^	17.6 (3)	C3—C2—C5—C6	−130.8 (4)
K2^xii^—N4—N5—K1^xii^	102.65 (9)	N4—N5—C6—C5	1.7 (4)
C4—N4—N5—K2^xii^	125.7 (3)	K1^x^—N5—C6—C5	−148.0 (2)
K2^xi^—N4—N5—K2^xii^	−85.1 (2)	K1^xii^—N5—C6—C5	110.3 (3)
K1^xii^—N4—N5—K2^xii^	−102.65 (9)	K2^xii^—N5—C6—C5	−59.2 (4)
K1^x^—O3—N6—O4	178.3 (2)	N4—N5—C6—N6	−178.9 (3)
K2^v^—O3—N6—O4	11.6 (3)	K1^x^—N5—C6—N6	31.5 (3)
K1^x^—O3—N6—C6	−2.1 (4)	K1^xii^—N5—C6—N6	−70.3 (3)
K2^v^—O3—N6—C6	−168.8 (2)	K2^xii^—N5—C6—N6	120.2 (3)
K1^x^—O3—N6—K2^v^	166.7 (2)	C4—C5—C6—N5	−1.0 (4)
K2^v^—O4—N6—O3	−13.3 (3)	C2—C5—C6—N5	174.8 (3)
K2^i^—O4—N6—O3	73.7 (3)	C4—C5—C6—N6	179.7 (3)
K2^v^—O4—N6—C6	167.1 (2)	C2—C5—C6—N6	−4.5 (7)
K2^i^—O4—N6—C6	−105.9 (2)	O3—N6—C6—N5	−20.5 (4)
K2^i^—O4—N6—K2^v^	87.01 (13)	O4—N6—C6—N5	159.2 (3)
N2—N1—C1—C2	0.6 (4)	K2^v^—N6—C6—N5	−146.9 (7)
K1^viii^—N1—C1—C2	−148.5 (2)	O3—N6—C6—C5	158.9 (3)
K1—N1—C1—C2	117.4 (3)	O4—N6—C6—C5	−21.5 (5)
N1—C1—C2—C3	0.1 (4)	K2^v^—N6—C6—C5	32.4 (11)

Symmetry codes: (i) −*x*+1/2, *y*, *z*−1/2; (ii) *x*, *y*−1, *z*; (iii) *x*+1/2, −*y*−1, *z*; (iv) *x*+1/2, −*y*, *z*; (v) −*x*+1/2, *y*−1, *z*−1/2; (vi) −*x*+1/2, *y*+1, *z*+1/2; (vii) *x*+1/2, −*y*+1, *z*; (viii) *x*, *y*+1, *z*; (ix) −*x*+1/2, *y*, *z*+1/2; (x) *x*−1/2, −*y*−1, *z*; (xi) *x*−1/2, −*y*+1, *z*; (xii) *x*−1/2, −*y*, *z*.

### Poly[[µ-3,3'-dinitro-4,4'-bipyrazole-1,1'-diido]dipotassium] (1) Hydrogen-bond geometry (Å, º)

**Table:**

*D*—H···*A*	*D*—H	H···*A*	*D*···*A*	*D*—H···*A*
C4—H4···O3^xiii^	0.94	2.62	3.352 (4)	135

Symmetry code: (xiii) −*x*, −*y*, *z*+1/2.

### Poly[[µ-3,3'-dinitro-4,4'-bipyrazole-1,1'-diido]dirubidium] (2) Crystal data

**Table:**

[Rb_2_(C_6_H_2_N_6_O_4_)]	*F*(000) = 744
*M**_r_* = 393.08	*D*_x_ = 2.495 Mg m^−^^3^
Monoclinic, *P*2_1_/*n*	Cu *K*α radiation, λ = 1.54186 Å
*a* = 3.9669 (2) Å	Cell parameters from 10655 reflections
*b* = 14.2020 (4) Å	θ = 3.9–79.9°
*c* = 18.6685 (6) Å	µ = 12.38 mm^−^^1^
β = 95.776 (3)°	*T* = 173 K
*V* = 1046.40 (7) Å^3^	Prism, red
*Z* = 4	0.05 × 0.03 × 0.03 mm

### Poly[[µ-3,3'-dinitro-4,4'-bipyrazole-1,1'-diido]dirubidium] (2) Data collection

**Table:**

Stoe Stadivari diffractometer	2234 independent reflections
Radiation source: GeniX 3D HF Cu	2156 reflections with *I* > 2σ(*I*)
Graded multilayer mirror monochromator	*R*_int_ = 0.030
Detector resolution: 5.81 pixels mm^-1^	θ_max_ = 79.9°, θ_min_ = 3.9°
rotation method, ω scans	*h* = −4→5
Absorption correction: multi-scan (Stoe *LANA*; Koziskova *et al.*, 2016)	*k* = −16→18
*T*_min_ = 0.469, *T*_max_ = 0.661	*l* = −23→13
10655 measured reflections	

### Poly[[µ-3,3'-dinitro-4,4'-bipyrazole-1,1'-diido]dirubidium] (2) Refinement

**Table:**

Refinement on *F*^2^	Secondary atom site location: difference Fourier map
Least-squares matrix: full	Hydrogen site location: inferred from neighbouring sites
*R*[*F*^2^ > 2σ(*F*^2^)] = 0.048	H-atom parameters constrained
*wR*(*F*^2^) = 0.148	*w* = 1/[σ^2^(*F*_o_^2^) + (0.1033*P*)^2^ + 1.4588*P*] where *P* = (*F*_o_^2^ + 2*F*_c_^2^)/3
*S* = 1.14	(Δ/σ)_max_ < 0.001
2234 reflections	Δρ_max_ = 0.86 e Å^−^^3^
164 parameters	Δρ_min_ = −0.72 e Å^−^^3^
0 restraints	Extinction correction: SHELXL-2019/2 (Sheldrick 2015), Fc^*^=kFc[1+0.001xFc^2^λ^3^/sin(2θ)]^-1/4^
Primary atom site location: structure-invariant direct methods	Extinction coefficient: 0.0053 (6)

### Poly[[µ-3,3'-dinitro-4,4'-bipyrazole-1,1'-diido]dirubidium] (2) Special details

**Table:**

Geometry. All esds (except the esd in the dihedral angle between two l.s. planes) are estimated using the full covariance matrix. The cell esds are taken into account individually in the estimation of esds in distances, angles and torsion angles; correlations between esds in cell parameters are only used when they are defined by crystal symmetry. An approximate (isotropic) treatment of cell esds is used for estimating esds involving l.s. planes.

### Poly[[µ-3,3'-dinitro-4,4'-bipyrazole-1,1'-diido]dirubidium] (2) Fractional atomic coordinates and isotropic or equivalent isotropic displacement parameters (Å^2^)

**Table:**

	*x*	*y*	*z*	*U*_iso_*/*U*_eq_	
Rb1	0.45494 (11)	0.14140 (3)	0.16029 (2)	0.0392 (2)	
Rb2	0.58119 (11)	0.41180 (3)	0.12337 (2)	0.0362 (2)	
O1	0.7633 (14)	0.0654 (3)	0.4780 (2)	0.0632 (13)	
O2	0.4337 (10)	0.0869 (2)	0.38057 (19)	0.0455 (9)	
O3	0.5123 (11)	0.5029 (3)	0.2664 (2)	0.0462 (8)	
O4	0.6039 (13)	0.4655 (3)	0.3786 (2)	0.0551 (10)	
N1	0.4128 (11)	0.3078 (3)	0.5487 (2)	0.0402 (9)	
N2	0.5224 (10)	0.2195 (3)	0.5384 (2)	0.0365 (8)	
N3	0.5540 (11)	0.1145 (3)	0.4407 (2)	0.0393 (8)	
N4	−0.0293 (10)	0.2675 (3)	0.2376 (2)	0.0380 (8)	
N5	0.1348 (11)	0.3503 (3)	0.2363 (2)	0.0369 (8)	
N6	0.4738 (11)	0.4480 (3)	0.3171 (2)	0.0377 (8)	
C1	0.2944 (14)	0.3447 (4)	0.4844 (3)	0.0402 (10)	
H1	0.208345	0.406910	0.477873	0.048*	
C2	0.3144 (12)	0.2798 (3)	0.4285 (2)	0.0357 (9)	
C3	0.4585 (11)	0.2026 (3)	0.4670 (2)	0.0345 (9)	
C4	0.0201 (13)	0.2325 (3)	0.3057 (2)	0.0376 (9)	
H4	−0.068538	0.174024	0.319981	0.045*	
C5	0.2154 (11)	0.2923 (3)	0.3518 (2)	0.0344 (9)	
C6	0.2779 (12)	0.3658 (3)	0.3039 (2)	0.0348 (9)	

### Poly[[µ-3,3'-dinitro-4,4'-bipyrazole-1,1'-diido]dirubidium] (2) Atomic displacement parameters (Å^2^)

**Table:**

	*U*^11^	*U*^22^	*U*^33^	*U*^12^	*U*^13^	*U*^23^
Rb1	0.0390 (3)	0.0381 (3)	0.0404 (3)	−0.00137 (15)	0.0037 (2)	0.00171 (16)
Rb2	0.0402 (3)	0.0377 (3)	0.0304 (3)	−0.00063 (15)	0.0022 (2)	0.00187 (14)
O1	0.084 (3)	0.052 (2)	0.048 (2)	0.029 (2)	−0.023 (2)	−0.0114 (18)
O2	0.059 (2)	0.0404 (19)	0.0355 (19)	0.0052 (14)	−0.0054 (17)	−0.0062 (13)
O3	0.062 (2)	0.0378 (17)	0.0398 (19)	−0.0062 (15)	0.0081 (16)	0.0001 (14)
O4	0.081 (3)	0.048 (2)	0.0340 (18)	−0.0181 (19)	−0.0040 (17)	0.0008 (15)
N1	0.049 (2)	0.041 (2)	0.0309 (19)	0.0016 (17)	0.0047 (16)	−0.0012 (15)
N2	0.0413 (19)	0.0389 (19)	0.0291 (17)	0.0016 (15)	0.0028 (14)	0.0023 (15)
N3	0.042 (2)	0.039 (2)	0.036 (2)	0.0027 (17)	0.0001 (16)	−0.0006 (16)
N4	0.040 (2)	0.043 (2)	0.0309 (18)	−0.0046 (16)	0.0015 (15)	−0.0033 (15)
N5	0.038 (2)	0.042 (2)	0.0306 (19)	0.0019 (15)	−0.0001 (15)	0.0029 (14)
N6	0.047 (2)	0.0336 (18)	0.0325 (19)	0.0019 (16)	0.0056 (16)	−0.0013 (15)
C1	0.052 (3)	0.039 (2)	0.029 (2)	0.006 (2)	0.0005 (19)	−0.0017 (17)
C2	0.042 (2)	0.033 (2)	0.032 (2)	0.0000 (17)	0.0021 (17)	0.0007 (17)
C3	0.036 (2)	0.036 (2)	0.030 (2)	−0.0015 (17)	0.0008 (16)	0.0014 (16)
C4	0.047 (2)	0.037 (2)	0.029 (2)	0.0017 (18)	0.0019 (18)	0.0003 (17)
C5	0.037 (2)	0.036 (2)	0.030 (2)	0.0036 (16)	0.0013 (16)	0.0004 (16)
C6	0.038 (2)	0.038 (2)	0.029 (2)	0.0017 (16)	0.0037 (17)	0.0017 (16)

### Poly[[µ-3,3'-dinitro-4,4'-bipyrazole-1,1'-diido]dirubidium] (2) Geometric parameters (Å, º)

**Table:**

Rb1—N1^i^	2.930 (4)	O2—N3	1.238 (5)
Rb1—N4^ii^	2.980 (4)	O3—N6	1.247 (5)
Rb1—N1^iii^	2.987 (4)	O4—N6	1.235 (6)
Rb1—N4	3.088 (4)	N1—N2	1.347 (6)
Rb1—O3^iv^	3.103 (4)	N1—C1	1.350 (6)
Rb1—O3^v^	3.113 (4)	N2—C3	1.353 (6)
Rb1—O4^iv^	3.175 (4)	N3—C3	1.410 (6)
Rb1—O4^v^	3.370 (5)	N4—N5	1.345 (6)
Rb2—O1^i^	2.893 (4)	N4—C4	1.361 (6)
Rb2—O1^vi^	2.993 (4)	N5—C6	1.348 (6)
Rb2—O3	3.004 (4)	N6—C6	1.410 (6)
Rb2—N5	3.016 (4)	C1—C2	1.401 (6)
Rb2—N5^ii^	3.017 (4)	C1—H1	0.9500
Rb2—N2^iii^	3.101 (4)	C2—C3	1.401 (6)
Rb2—O2^vi^	3.150 (4)	C2—C5	1.456 (6)
Rb2—N2^i^	3.194 (4)	C4—C5	1.389 (6)
Rb2—O2^vii^	3.214 (4)	C4—H4	0.9500
O1—N3	1.243 (6)	C5—C6	1.413 (6)
			
N1^i^—Rb1—N4^ii^	127.98 (12)	N6—O3—Rb1^vii^	86.4 (3)
N1^i^—Rb1—N1^iii^	84.19 (10)	Rb2—O3—Rb1^vii^	140.74 (14)
N4^ii^—Rb1—N1^iii^	75.92 (11)	Rb1^vi^—O3—Rb1^vii^	79.32 (9)
N1^i^—Rb1—N4	75.11 (11)	N6—O4—Rb1^vi^	97.8 (3)
N4^ii^—Rb1—N4	81.61 (10)	N6—O4—Rb1^vii^	75.3 (3)
N1^iii^—Rb1—N4	129.52 (11)	Rb1^vi^—O4—Rb1^vii^	74.55 (8)
N1^i^—Rb1—O3^iv^	152.66 (11)	N2—N1—C1	108.9 (4)
N4^ii^—Rb1—O3^iv^	76.33 (11)	N2—N1—Rb1^viii^	96.1 (3)
N1^iii^—Rb1—O3^iv^	91.48 (11)	C1—N1—Rb1^viii^	134.5 (3)
N4—Rb1—O3^iv^	126.14 (10)	N2—N1—Rb1^ix^	123.5 (3)
N1^i^—Rb1—O3^v^	92.37 (11)	C1—N1—Rb1^ix^	110.2 (3)
N4^ii^—Rb1—O3^v^	125.25 (10)	Rb1^viii^—N1—Rb1^ix^	84.18 (10)
N1^iii^—Rb1—O3^v^	152.88 (10)	N1—N2—C3	105.9 (4)
N4—Rb1—O3^v^	74.65 (10)	N1—N2—Rb2^ix^	106.3 (3)
O3^iv^—Rb1—O3^v^	79.32 (9)	C3—N2—Rb2^ix^	109.8 (3)
N1^i^—Rb1—O4^iv^	114.40 (11)	N1—N2—Rb2^viii^	133.7 (3)
N4^ii^—Rb1—O4^iv^	102.48 (12)	C3—N2—Rb2^viii^	116.0 (3)
N1^iii^—Rb1—O4^iv^	69.07 (12)	Rb2^ix^—N2—Rb2^viii^	78.11 (9)
N4—Rb1—O4^iv^	161.07 (11)	N1—N2—Rb1^viii^	60.4 (2)
O3^iv^—Rb1—O4^iv^	40.13 (9)	C3—N2—Rb1^viii^	143.7 (3)
O3^v^—Rb1—O4^iv^	88.25 (11)	Rb2^ix^—N2—Rb1^viii^	106.49 (11)
N1^i^—Rb1—O4^v^	67.01 (10)	Rb2^viii^—N2—Rb1^viii^	74.02 (8)
N4^ii^—Rb1—O4^v^	162.62 (10)	O2—N3—O1	120.4 (4)
N1^iii^—Rb1—O4^v^	117.51 (11)	O2—N3—C3	120.3 (4)
N4—Rb1—O4^v^	95.96 (11)	O1—N3—C3	119.3 (4)
O3^iv^—Rb1—O4^v^	91.67 (10)	O2—N3—Rb2^iv^	64.1 (2)
O3^v^—Rb1—O4^v^	38.57 (9)	O1—N3—Rb2^iv^	56.8 (3)
O4^iv^—Rb1—O4^v^	74.55 (8)	C3—N3—Rb2^iv^	170.9 (3)
O1^i^—Rb2—O1^vi^	54.79 (14)	N5—N4—C4	108.2 (4)
O1^i^—Rb2—O3	135.69 (14)	N5—N4—Rb1^x^	145.7 (3)
O1^vi^—Rb2—O3	106.93 (12)	C4—N4—Rb1^x^	105.0 (3)
O1^i^—Rb2—N5	117.20 (13)	N5—N4—Rb1	100.0 (3)
O1^vi^—Rb2—N5	145.70 (14)	C4—N4—Rb1	100.8 (3)
O3—Rb2—N5	52.47 (11)	Rb1^x^—N4—Rb1	81.61 (10)
O1^i^—Rb2—N5^ii^	154.94 (14)	N5—N4—Rb2^x^	68.6 (2)
O1^vi^—Rb2—N5^ii^	118.11 (12)	C4—N4—Rb2^x^	149.5 (3)
O3—Rb2—N5^ii^	68.18 (11)	Rb1^x^—N4—Rb2^x^	78.61 (9)
N5—Rb2—N5^ii^	82.23 (10)	Rb1—N4—Rb2^x^	109.73 (11)
O1^i^—Rb2—N2^iii^	78.61 (14)	N4—N5—C6	106.6 (4)
O1^vi^—Rb2—N2^iii^	87.26 (14)	N4—N5—Rb2	125.8 (3)
O3—Rb2—N2^iii^	145.10 (11)	C6—N5—Rb2	112.7 (3)
N5—Rb2—N2^iii^	125.79 (11)	N4—N5—Rb2^x^	86.9 (3)
N5^ii^—Rb2—N2^iii^	76.98 (11)	C6—N5—Rb2^x^	144.6 (3)
O1^i^—Rb2—O2^vi^	95.71 (10)	Rb2—N5—Rb2^x^	82.23 (10)
O1^vi^—Rb2—O2^vi^	40.92 (10)	O4—N6—O3	120.5 (4)
O3—Rb2—O2^vi^	77.95 (10)	O4—N6—C6	120.1 (4)
N5—Rb2—O2^vi^	130.39 (11)	O3—N6—C6	119.4 (4)
N5^ii^—Rb2—O2^vi^	80.87 (10)	O4—N6—Rb1^vii^	83.4 (3)
N2^iii^—Rb2—O2^vi^	94.89 (11)	O3—N6—Rb1^vii^	71.3 (3)
O1^i^—Rb2—N2^i^	51.99 (11)	C6—N6—Rb1^vii^	115.6 (3)
O1^vi^—Rb2—N2^i^	106.77 (10)	O4—N6—Rb1^vi^	62.1 (3)
O3—Rb2—N2^i^	124.90 (11)	O3—N6—Rb1^vi^	58.8 (2)
N5—Rb2—N2^i^	75.59 (11)	C6—N6—Rb1^vi^	173.1 (3)
N5^ii^—Rb2—N2^i^	126.74 (11)	Rb1^vii^—N6—Rb1^vi^	70.77 (8)
N2^iii^—Rb2—N2^i^	78.11 (9)	N1—C1—C2	111.5 (4)
O2^vi^—Rb2—N2^i^	147.62 (9)	N1—C1—H1	124.3
O1^i^—Rb2—O2^vii^	71.10 (13)	C2—C1—H1	124.3
O1^vi^—Rb2—O2^vii^	65.94 (13)	C1—C2—C3	100.5 (4)
O3—Rb2—O2^vii^	64.71 (10)	C1—C2—C5	128.5 (4)
N5—Rb2—O2^vii^	79.84 (10)	C3—C2—C5	131.0 (4)
N5^ii^—Rb2—O2^vii^	131.02 (10)	N2—C3—C2	113.2 (4)
N2^iii^—Rb2—O2^vii^	147.53 (10)	N2—C3—N3	118.1 (4)
O2^vi^—Rb2—O2^vii^	77.11 (8)	C2—C3—N3	128.7 (4)
N2^i^—Rb2—O2^vii^	91.88 (10)	N4—C4—C5	112.0 (4)
N3—O1—Rb2^viii^	131.8 (3)	N4—C4—H4	124.0
N3—O1—Rb2^iv^	102.9 (3)	C5—C4—H4	124.0
Rb2^viii^—O1—Rb2^iv^	125.21 (14)	C4—C5—C6	100.5 (4)
N3—O2—Rb2^iv^	95.2 (3)	C4—C5—C2	127.8 (4)
N3—O2—Rb2^v^	116.9 (3)	C6—C5—C2	131.7 (4)
Rb2^iv^—O2—Rb2^v^	77.11 (8)	N5—C6—N6	118.2 (4)
N6—O3—Rb2	115.7 (3)	N5—C6—C5	112.8 (4)
N6—O3—Rb1^vi^	101.0 (3)	N6—C6—C5	129.0 (4)
Rb2—O3—Rb1^vi^	123.10 (13)		
			
C1—N1—N2—C3	−1.9 (5)	Rb1^ix^—N1—C1—C2	−136.8 (4)
Rb1^viii^—N1—N2—C3	−143.3 (3)	N1—C1—C2—C5	−179.6 (5)
Rb1^ix^—N1—N2—C3	129.7 (3)	N1—N2—C3—C2	1.6 (5)
C1—N1—N2—Rb2^ix^	−118.5 (4)	Rb2^ix^—N2—C3—C2	115.9 (4)
Rb1^viii^—N1—N2—Rb2^ix^	100.03 (16)	Rb2^viii^—N2—C3—C2	−158.0 (3)
Rb1^ix^—N1—N2—Rb2^ix^	13.0 (4)	Rb1^viii^—N2—C3—C2	−59.7 (6)
C1—N1—N2—Rb2^viii^	152.5 (4)	N1—N2—C3—N3	179.0 (4)
Rb1^viii^—N1—N2—Rb2^viii^	11.1 (4)	Rb2^ix^—N2—C3—N3	−66.7 (4)
Rb1^ix^—N1—N2—Rb2^viii^	−76.0 (4)	Rb2^viii^—N2—C3—N3	19.4 (5)
C1—N1—N2—Rb1^viii^	141.4 (4)	Rb1^viii^—N2—C3—N3	117.7 (5)
Rb1^ix^—N1—N2—Rb1^viii^	−87.0 (3)	C1—C2—C3—N2	−0.7 (6)
Rb2^iv^—O2—N3—O1	−8.1 (5)	C5—C2—C3—N2	178.4 (5)
Rb2^v^—O2—N3—O1	70.1 (6)	C1—C2—C3—N3	−177.8 (5)
Rb2^iv^—O2—N3—C3	171.0 (4)	C5—C2—C3—N3	1.3 (9)
Rb2^v^—O2—N3—C3	−110.9 (4)	O2—N3—C3—N2	163.0 (4)
Rb2^v^—O2—N3—Rb2^iv^	78.17 (18)	O1—N3—C3—N2	−17.9 (7)
Rb2^viii^—O1—N3—O2	−174.8 (4)	O2—N3—C3—C2	−20.0 (8)
Rb2^iv^—O1—N3—O2	8.7 (6)	O1—N3—C3—C2	159.1 (5)
Rb2^viii^—O1—N3—C3	6.1 (8)	N5—N4—C4—C5	0.9 (6)
Rb2^iv^—O1—N3—C3	−170.4 (3)	Rb1^x^—N4—C4—C5	−170.6 (3)
Rb2^viii^—O1—N3—Rb2^iv^	176.5 (6)	Rb1—N4—C4—C5	105.3 (4)
C4—N4—N5—C6	−1.0 (5)	Rb2^x^—N4—C4—C5	−77.6 (7)
Rb1^x^—N4—N5—C6	164.3 (4)	N5—N4—C4—Rb1^x^	171.5 (4)
Rb1—N4—N5—C6	−106.0 (3)	Rb1—N4—C4—Rb1^x^	−84.11 (17)
Rb2^x^—N4—N5—C6	146.6 (4)	Rb2^x^—N4—C4—Rb1^x^	93.1 (6)
C4—N4—N5—Rb2	134.2 (3)	N5—N4—C4—Rb1	−104.4 (4)
Rb1^x^—N4—N5—Rb2	−60.5 (6)	Rb1^x^—N4—C4—Rb1	84.11 (17)
Rb1—N4—N5—Rb2	29.3 (3)	Rb2^x^—N4—C4—Rb1	177.2 (7)
Rb2^x^—N4—N5—Rb2	−78.1 (2)	N4—C4—C5—C6	−0.4 (5)
C4—N4—N5—Rb2^x^	−147.7 (3)	N4—C4—C5—C2	178.5 (4)
Rb1^x^—N4—N5—Rb2^x^	17.6 (5)	C1—C2—C5—C4	−131.9 (6)
Rb1—N4—N5—Rb2^x^	107.40 (13)	C3—C2—C5—C4	49.3 (8)
Rb1^vi^—O4—N6—O3	−8.0 (5)	C1—C2—C5—C6	46.6 (8)
Rb1^vii^—O4—N6—O3	63.7 (4)	C3—C2—C5—C6	−132.2 (6)
Rb1^vi^—O4—N6—C6	172.5 (4)	N4—N5—C6—N6	178.6 (4)
Rb1^vii^—O4—N6—C6	−115.8 (4)	Rb2—N5—C6—N6	36.8 (5)
Rb1^vi^—O4—N6—Rb1^vii^	−71.71 (13)	Rb2^x^—N5—C6—N6	−72.7 (7)
Rb1^vii^—O4—N6—Rb1^vi^	71.71 (13)	N4—N5—C6—C5	0.9 (5)
Rb2—O3—N6—O4	143.7 (4)	Rb2—N5—C6—C5	−140.9 (3)
Rb1^vi^—O3—N6—O4	8.3 (5)	Rb2^x^—N5—C6—C5	109.6 (5)
Rb1^vii^—O3—N6—O4	−70.1 (5)	O4—N6—C6—N5	178.7 (5)
Rb2—O3—N6—C6	−36.8 (5)	O3—N6—C6—N5	−0.8 (7)
Rb1^vi^—O3—N6—C6	−172.2 (3)	Rb1^vii^—N6—C6—N5	81.2 (4)
Rb1^vii^—O3—N6—C6	109.4 (4)	O4—N6—C6—C5	−4.0 (8)
Rb2—O3—N6—Rb1^vii^	−146.2 (3)	O3—N6—C6—C5	176.5 (5)
Rb1^vi^—O3—N6—Rb1^vii^	78.39 (12)	Rb1^vii^—N6—C6—C5	−101.5 (5)
Rb2—O3—N6—Rb1^vi^	135.4 (3)	C4—C5—C6—N5	−0.3 (5)
Rb1^vii^—O3—N6—Rb1^vi^	−78.39 (12)	C2—C5—C6—N5	−179.1 (5)
N2—N1—C1—C2	1.5 (6)	C4—C5—C6—N6	−177.7 (5)
Rb1^viii^—N1—C1—C2	121.1 (4)	C2—C5—C6—N6	3.5 (8)

Symmetry codes: (i) *x*−1/2, −*y*+1/2, *z*−1/2; (ii) *x*+1, *y*, *z*; (iii) *x*+1/2, −*y*+1/2, *z*−1/2; (iv) −*x*+3/2, *y*−1/2, −*z*+1/2; (v) −*x*+1/2, *y*−1/2, −*z*+1/2; (vi) −*x*+3/2, *y*+1/2, −*z*+1/2; (vii) −*x*+1/2, *y*+1/2, −*z*+1/2; (viii) *x*+1/2, −*y*+1/2, *z*+1/2; (ix) *x*−1/2, −*y*+1/2, *z*+1/2; (x) *x*−1, *y*, *z*.

### Poly[[µ-3,3'-dinitro-4,4'-bipyrazole-1,1'-diido]dirubidium] (2) Hydrogen-bond geometry (Å, º)

**Table:**

*D*—H···*A*	*D*—H	H···*A*	*D*···*A*	*D*—H···*A*
C4—H4···O2^x^	0.95	2.68	3.506 (6)	146

Symmetry code: (x) *x*−1, *y*, *z*.

### Poly[[[µ-3,3'-dinitro-4,4'-bipyrazole-1,1'-diido]dicaesium] monohydrate] (3) Crystal data

**Table:**

[Cs_2_(C_6_H_2_N_6_O_4_)]·H_2_O	*F*(000) = 464
*M**_r_* = 505.97	*D*_x_ = 2.733 Mg m^−^^3^
Monoclinic, *P*2_1_	Mo *K*α radiation, λ = 0.71073 Å
*a* = 9.7388 (7) Å	Cell parameters from 4971 reflections
*b* = 6.9551 (3) Å	θ = 2.4–27.1°
*c* = 10.2950 (7) Å	µ = 5.96 mm^−^^1^
β = 118.152 (8)°	*T* = 213 K
*V* = 614.83 (8) Å^3^	Prism, red
*Z* = 2	0.20 × 0.17 × 0.14 mm

### Poly[[[µ-3,3'-dinitro-4,4'-bipyrazole-1,1'-diido]dicaesium] monohydrate] (3) Data collection

**Table:**

Stoe Image plate diffraction system-2T diffractometer	2376 reflections with *I* > 2σ(*I*)
Radiation source: fine-focus sealed tube	*R*_int_ = 0.020
φ oscillation scans	θ_max_ = 27.1°, θ_min_ = 2.4°
Absorption correction: numerical [X-RED (Stoe & Cie, 2001) and X-SHAPE (Stoe & Cie, 1999)]	*h* = −12→12
*T*_min_ = 0.218, *T*_max_ = 0.245	*k* = −8→8
4971 measured reflections	*l* = −13→13
2554 independent reflections	

### Poly[[[µ-3,3'-dinitro-4,4'-bipyrazole-1,1'-diido]dicaesium] monohydrate] (3) Refinement

**Table:**

Refinement on *F*^2^	Secondary atom site location: difference Fourier map
Least-squares matrix: full	Hydrogen site location: mixed
*R*[*F*^2^ > 2σ(*F*^2^)] = 0.020	H-atom parameters constrained
*wR*(*F*^2^) = 0.045	*w* = 1/[σ^2^(*F*_o_^2^) + (0.0271*P*)^2^] where *P* = (*F*_o_^2^ + 2*F*_c_^2^)/3
*S* = 0.99	(Δ/σ)_max_ < 0.001
2554 reflections	Δρ_max_ = 0.93 e Å^−^^3^
173 parameters	Δρ_min_ = −1.17 e Å^−^^3^
1 restraint	Absolute structure: Refined as an inversion twin
Primary atom site location: structure-invariant direct methods	Absolute structure parameter: 0.44 (3)

### Poly[[[µ-3,3'-dinitro-4,4'-bipyrazole-1,1'-diido]dicaesium] monohydrate] (3) Special details

**Table:**

Geometry. All esds (except the esd in the dihedral angle between two l.s. planes) are estimated using the full covariance matrix. The cell esds are taken into account individually in the estimation of esds in distances, angles and torsion angles; correlations between esds in cell parameters are only used when they are defined by crystal symmetry. An approximate (isotropic) treatment of cell esds is used for estimating esds involving l.s. planes.
Refinement. Refined as a 2-component inversion twin.

### Poly[[[µ-3,3'-dinitro-4,4'-bipyrazole-1,1'-diido]dicaesium] monohydrate] (3) Fractional atomic coordinates and isotropic or equivalent isotropic displacement parameters (Å^2^)

**Table:**

	*x*	*y*	*z*	*U*_iso_*/*U*_eq_	
Cs1	0.37557 (4)	−0.01332 (6)	0.83234 (4)	0.02622 (11)	
Cs2	0.28594 (4)	0.21708 (6)	0.40418 (4)	0.02770 (11)	
O1	0.2933 (5)	0.6346 (8)	0.9720 (6)	0.0378 (13)	
O2	0.0596 (6)	0.6050 (9)	0.7982 (7)	0.0550 (19)	
O3	−0.0531 (7)	0.3586 (11)	0.4159 (6)	0.058 (2)	
O4	0.1297 (5)	0.2870 (10)	0.6288 (6)	0.0460 (17)	
N1	0.2513 (6)	0.0986 (9)	1.0846 (6)	0.0256 (13)	
N2	0.2885 (6)	0.2776 (9)	1.0688 (5)	0.0225 (12)	
N3	0.1744 (6)	0.5375 (9)	0.9019 (6)	0.0281 (15)	
N4	−0.3494 (6)	0.2071 (11)	0.5665 (6)	0.0302 (13)	
N5	−0.2719 (5)	0.2515 (10)	0.4893 (5)	0.0270 (13)	
N6	−0.0093 (6)	0.3032 (10)	0.5416 (6)	0.0279 (13)	
C1	0.1092 (7)	0.0551 (11)	0.9684 (7)	0.0245 (14)	
H1	0.057551	−0.063088	0.955557	0.029*	
C2	0.0521 (5)	0.2089 (11)	0.8725 (6)	0.0177 (11)	
C3	0.1704 (7)	0.3440 (10)	0.9432 (7)	0.0211 (13)	
C4	−0.2440 (7)	0.1895 (11)	0.7100 (7)	0.0241 (15)	
H4	−0.269184	0.160356	0.785344	0.029*	
C5	−0.0932 (6)	0.2199 (11)	0.7323 (6)	0.0201 (11)	
C6	−0.1201 (6)	0.2587 (10)	0.5895 (6)	0.0218 (14)	
O1W	0.4552 (6)	0.4093 (9)	0.7600 (7)	0.0527 (18)	
H1W	0.546627	0.455957	0.802129	0.079*	
H2W	0.395147	0.489868	0.696509	0.079*	

### Poly[[[µ-3,3'-dinitro-4,4'-bipyrazole-1,1'-diido]dicaesium] monohydrate] (3) Atomic displacement parameters (Å^2^)

**Table:**

	*U*^11^	*U*^22^	*U*^33^	*U*^12^	*U*^13^	*U*^23^
Cs1	0.01693 (17)	0.0311 (3)	0.02732 (19)	0.00105 (17)	0.00772 (14)	−0.00028 (19)
Cs2	0.01801 (17)	0.0343 (3)	0.0277 (2)	−0.00397 (19)	0.00824 (14)	0.00034 (19)
O1	0.021 (2)	0.026 (3)	0.051 (3)	−0.008 (2)	0.005 (2)	0.002 (2)
O2	0.033 (3)	0.026 (4)	0.067 (4)	−0.001 (2)	−0.010 (3)	0.016 (3)
O3	0.035 (3)	0.105 (6)	0.031 (3)	0.006 (3)	0.012 (2)	0.022 (3)
O4	0.017 (2)	0.081 (5)	0.040 (3)	0.005 (2)	0.013 (2)	0.022 (3)
N1	0.024 (3)	0.026 (4)	0.026 (3)	0.000 (2)	0.011 (2)	0.003 (2)
N2	0.018 (2)	0.025 (4)	0.020 (2)	0.002 (2)	0.006 (2)	0.000 (2)
N3	0.017 (2)	0.027 (5)	0.031 (3)	0.002 (2)	0.003 (2)	0.004 (2)
N4	0.016 (2)	0.032 (4)	0.037 (3)	−0.005 (3)	0.007 (2)	−0.004 (3)
N5	0.015 (2)	0.034 (4)	0.025 (3)	0.004 (2)	0.0031 (19)	−0.003 (3)
N6	0.023 (3)	0.034 (4)	0.024 (3)	0.005 (2)	0.009 (2)	0.005 (2)
C1	0.020 (3)	0.029 (4)	0.026 (3)	−0.003 (3)	0.013 (3)	0.000 (3)
C2	0.010 (2)	0.021 (3)	0.021 (2)	0.001 (3)	0.0060 (19)	−0.001 (3)
C3	0.020 (3)	0.016 (4)	0.025 (3)	0.000 (2)	0.008 (3)	0.000 (3)
C4	0.019 (3)	0.021 (4)	0.032 (3)	−0.004 (3)	0.012 (2)	−0.002 (3)
C5	0.012 (2)	0.020 (3)	0.024 (3)	0.002 (3)	0.005 (2)	−0.004 (3)
C6	0.013 (2)	0.023 (4)	0.026 (3)	0.003 (2)	0.006 (2)	−0.001 (3)
O1W	0.024 (3)	0.049 (5)	0.065 (4)	−0.010 (3)	0.005 (3)	0.006 (3)

### Poly[[[µ-3,3'-dinitro-4,4'-bipyrazole-1,1'-diido]dicaesium] monohydrate] (3) Geometric parameters (Å, º)

**Table:**

Cs1—O1^i^	3.064 (5)	O3—N6	1.218 (7)
Cs1—O3^ii^	3.101 (6)	O4—N6	1.227 (7)
Cs1—O4	3.123 (5)	N1—N2	1.328 (8)
Cs1—O1^iii^	3.130 (6)	N1—C1	1.370 (8)
Cs1—O1W	3.215 (6)	N2—C3	1.344 (8)
Cs1—N2^i^	3.273 (5)	N3—C3	1.417 (9)
Cs1—N5^ii^	3.392 (6)	N4—C4	1.349 (8)
Cs1—Cg1	3.389 (3)	N4—N5	1.365 (8)
Cs1—O1W^i^	3.754 (7)	N5—C6	1.346 (7)
Cs2—O2^ii^	3.107 (5)	N6—C6	1.417 (8)
Cs2—N4^iv^	3.132 (5)	C1—C2	1.382 (10)
Cs2—N1^v^	3.251 (6)	C1—H1	0.9400
Cs2—O4	3.350 (5)	C2—C3	1.396 (9)
Cs2—O1W	3.496 (7)	C2—C5	1.471 (7)
Cs2—O3	3.502 (6)	C4—C5	1.391 (8)
Cs2—Cg2^ii^	3.474 (3)	C4—H4	0.9400
Cs2—Cg2^vi^	3.587 (3)	C5—C6	1.392 (8)
O1—N3	1.236 (7)	O1W—H1W	0.8500
O2—N3	1.219 (7)	O1W—H2W	0.8500
			
O1^i^—Cs1—O3^ii^	168.81 (16)	N6—O4—Cs2	102.2 (4)
O1^i^—Cs1—O4	116.91 (15)	Cs1—O4—Cs2	84.94 (13)
O3^ii^—Cs1—O4	58.76 (19)	N2—N1—C1	108.7 (5)
O1^i^—Cs1—O1^iii^	112.27 (11)	N2—N1—Cs2^ix^	89.0 (4)
O3^ii^—Cs1—O1^iii^	76.97 (17)	C1—N1—Cs2^ix^	121.3 (4)
O4—Cs1—O1^iii^	123.38 (13)	N1—N2—C3	106.3 (5)
O1^i^—Cs1—O1W	63.35 (14)	N1—N2—Cs1^vii^	132.0 (4)
O3^ii^—Cs1—O1W	109.44 (18)	C3—N2—Cs1^vii^	115.0 (4)
O4—Cs1—O1W	54.93 (14)	N1—N2—Cs2^ix^	68.6 (3)
O1^iii^—Cs1—O1W	164.51 (17)	C3—N2—Cs2^ix^	126.2 (4)
O1^i^—Cs1—N2^i^	49.99 (14)	Cs1^vii^—N2—Cs2^ix^	102.63 (13)
O3^ii^—Cs1—N2^i^	127.14 (16)	O2—N3—O1	121.4 (7)
O4—Cs1—N2^i^	147.49 (13)	O2—N3—C3	119.0 (6)
O1^iii^—Cs1—N2^i^	86.82 (14)	O1—N3—C3	119.6 (5)
O1W—Cs1—N2^i^	99.40 (14)	C4—N4—N5	108.3 (5)
O1^i^—Cs1—N5^ii^	122.67 (14)	C4—N4—Cs2^x^	132.2 (4)
O3^ii^—Cs1—N5^ii^	48.52 (14)	N5—N4—Cs2^x^	118.9 (3)
O4—Cs1—N5^ii^	83.50 (16)	C4—N4—Cs2^ii^	78.6 (5)
O1^iii^—Cs1—N5^ii^	92.77 (15)	N5—N4—Cs2^ii^	98.6 (4)
O1W—Cs1—N5^ii^	102.00 (16)	Cs2^x^—N4—Cs2^ii^	100.36 (17)
N2^i^—Cs1—N5^ii^	83.26 (13)	C4—N4—Cs2^vi^	89.2 (4)
O1^i^—Cs1—O1W^i^	62.76 (15)	N5—N4—Cs2^vi^	72.8 (4)
O3^ii^—Cs1—O1W^i^	128.32 (15)	Cs2^x^—N4—Cs2^vi^	97.47 (18)
O4—Cs1—O1W^i^	129.74 (14)	Cs2^ii^—N4—Cs2^vi^	162.17 (15)
O1^iii^—Cs1—O1W^i^	56.41 (14)	C6—N5—N4	105.7 (5)
O1W—Cs1—O1W^i^	111.30 (16)	C6—N5—Cs1^vi^	116.3 (4)
N2^i^—Cs1—O1W^i^	75.01 (13)	N4—N5—Cs1^vi^	133.3 (4)
N5^ii^—Cs1—O1W^i^	142.53 (14)	C6—N5—Cs2^vi^	85.1 (4)
O2^ii^—Cs2—N4^iv^	161.58 (18)	N4—N5—Cs2^vi^	84.9 (4)
O2^ii^—Cs2—N1^v^	72.28 (17)	Cs1^vi^—N5—Cs2^vi^	79.57 (14)
N4^iv^—Cs2—N1^v^	94.85 (14)	O3—N6—O4	121.3 (6)
O2^ii^—Cs2—O4	78.36 (17)	O3—N6—C6	119.8 (6)
N4^iv^—Cs2—O4	113.88 (13)	O4—N6—C6	118.9 (5)
N1^v^—Cs2—O4	150.63 (13)	N1—C1—C2	110.7 (6)
O2^ii^—Cs2—N4^vi^	113.52 (14)	N1—C1—H1	124.7
N4^iv^—Cs2—N4^vi^	82.18 (15)	C2—C1—H1	124.7
N1^v^—Cs2—N4^vi^	105.51 (14)	C1—C2—C3	101.2 (5)
O4—Cs2—N4^vi^	85.25 (15)	C1—C2—C5	128.1 (6)
O2^ii^—Cs2—O1W	128.63 (17)	C3—C2—C5	130.7 (6)
N4^iv^—Cs2—O1W	66.05 (14)	N2—C3—C2	113.1 (6)
N1^v^—Cs2—O1W	158.38 (13)	N2—C3—N3	118.9 (6)
O4—Cs2—O1W	50.52 (13)	C2—C3—N3	128.0 (6)
N4^vi^—Cs2—O1W	63.54 (14)	N4—C4—C5	111.5 (6)
O2^ii^—Cs2—O3	49.70 (17)	N4—C4—H4	124.3
N4^iv^—Cs2—O3	146.93 (15)	C5—C4—H4	124.3
N1^v^—Cs2—O3	117.19 (14)	Cs2^ii^—C4—H4	92.3
O4—Cs2—O3	36.14 (12)	Cs2^vi^—C4—H4	115.5
N4^vi^—Cs2—O3	81.51 (15)	C4—C5—C6	101.3 (5)
O1W—Cs2—O3	80.93 (14)	C4—C5—C2	127.3 (5)
O2^ii^—Cs2—N4^ii^	84.12 (14)	C6—C5—C2	131.3 (5)
N4^iv^—Cs2—N4^ii^	79.99 (14)	N5—C6—C5	113.2 (5)
N1^v^—Cs2—N4^ii^	76.47 (14)	N5—C6—N6	118.8 (5)
O4—Cs2—N4^ii^	101.65 (15)	C5—C6—N6	128.0 (5)
N4^vi^—Cs2—N4^ii^	162.17 (15)	Cs1—O1W—Cs2	81.21 (14)
O1W—Cs2—N4^ii^	108.21 (14)	Cs1—O1W—Cs1^vii^	84.20 (14)
O3—Cs2—N4^ii^	113.76 (15)	Cs2—O1W—Cs1^vii^	165.33 (19)
N3—O1—Cs1^vii^	127.3 (4)	Cs1—O1W—H1W	122.7
N3—O1—Cs1^viii^	122.1 (4)	Cs2—O1W—H1W	122.9
Cs1^vii^—O1—Cs1^viii^	98.42 (14)	Cs1^vii^—O1W—H1W	64.7
N3—O2—Cs2^vi^	160.1 (6)	Cs1—O1W—H2W	128.9
N6—O3—Cs1^vi^	132.6 (4)	Cs2—O1W—H2W	68.5
N6—O3—Cs2	94.9 (4)	Cs1^vii^—O1W—H2W	123.0
Cs1^vi^—O3—Cs2	131.08 (18)	H1W—O1W—H2W	108.4
N6—O4—Cs1	141.7 (5)		
			
C1—N1—N2—C3	−0.3 (7)	C1—C2—C3—N3	−175.8 (6)
Cs2^ix^—N1—N2—C3	−123.2 (4)	C5—C2—C3—N3	5.7 (11)
C1—N1—N2—Cs1^vii^	−149.2 (4)	O2—N3—C3—N2	−171.2 (7)
Cs2^ix^—N1—N2—Cs1^vii^	87.9 (4)	O1—N3—C3—N2	8.3 (9)
C1—N1—N2—Cs2^ix^	122.9 (5)	O2—N3—C3—C2	5.1 (10)
Cs2^vi^—O2—N3—O1	−129.3 (13)	O1—N3—C3—C2	−175.4 (6)
Cs2^vi^—O2—N3—C3	50.2 (19)	N5—N4—C4—C5	−0.5 (9)
Cs1^vii^—O1—N3—O2	−159.5 (6)	Cs2^x^—N4—C4—C5	−171.5 (5)
Cs1^viii^—O1—N3—O2	−25.5 (10)	Cs2^ii^—N4—C4—C5	94.8 (6)
Cs1^vii^—O1—N3—C3	21.0 (9)	Cs2^vi^—N4—C4—C5	−72.1 (6)
Cs1^viii^—O1—N3—C3	155.0 (4)	N5—N4—C4—Cs2^ii^	−95.3 (5)
C4—N4—N5—C6	0.3 (8)	Cs2^x^—N4—C4—Cs2^ii^	93.7 (6)
Cs2^x^—N4—N5—C6	172.6 (4)	Cs2^vi^—N4—C4—Cs2^ii^	−166.94 (14)
Cs2^ii^—N4—N5—C6	−80.5 (5)	N5—N4—C4—Cs2^vi^	71.6 (5)
Cs2^vi^—N4—N5—C6	83.5 (5)	Cs2^x^—N4—C4—Cs2^vi^	−99.3 (6)
C4—N4—N5—Cs1^vi^	−153.7 (5)	Cs2^ii^—N4—C4—Cs2^vi^	166.94 (14)
Cs2^x^—N4—N5—Cs1^vi^	18.6 (8)	N4—C4—C5—C6	0.6 (8)
Cs2^ii^—N4—N5—Cs1^vi^	125.5 (4)	Cs2^ii^—C4—C5—C6	81.9 (5)
Cs2^vi^—N4—N5—Cs1^vi^	−70.5 (5)	Cs2^vi^—C4—C5—C6	−64.2 (5)
C4—N4—N5—Cs2^vi^	−83.2 (6)	N4—C4—C5—C2	−178.6 (7)
Cs2^x^—N4—N5—Cs2^vi^	89.1 (4)	C1—C2—C5—C4	57.5 (11)
Cs2^ii^—N4—N5—Cs2^vi^	−163.97 (14)	C3—C2—C5—C4	−124.4 (8)
Cs1^vi^—O3—N6—O4	−168.9 (6)	C1—C2—C5—C6	−121.4 (9)
Cs2—O3—N6—O4	24.2 (8)	C3—C2—C5—C6	56.7 (11)
Cs1^vi^—O3—N6—C6	10.3 (11)	C1—C2—C5—Cs2^vi^	153.5 (5)
Cs2—O3—N6—C6	−156.7 (6)	C3—C2—C5—Cs2^vi^	−28.4 (7)
Cs1—O4—N6—O3	−123.2 (7)	C1—C2—C5—Cs2^ii^	−12.3 (7)
Cs2—O4—N6—O3	−25.9 (8)	C3—C2—C5—Cs2^ii^	165.8 (5)
Cs1—O4—N6—C6	57.6 (10)	N4—N5—C6—C5	0.1 (9)
Cs2—O4—N6—C6	155.0 (5)	Cs1^vi^—N5—C6—C5	159.3 (5)
N2—N1—C1—C2	0.7 (7)	Cs2^vi^—N5—C6—C5	83.4 (6)
Cs2^ix^—N1—C1—C2	101.4 (5)	N4—N5—C6—N6	−179.7 (6)
N1—C1—C2—C3	−0.8 (7)	Cs1^vi^—N5—C6—N6	−20.6 (8)
N1—C1—C2—C5	177.7 (5)	Cs2^vi^—N5—C6—N6	−96.4 (6)
N1—N2—C3—C2	−0.3 (7)	C4—C5—C6—N5	−0.4 (8)
Cs1^vii^—N2—C3—C2	154.7 (4)	C2—C5—C6—N5	178.7 (8)
Cs2^ix^—N2—C3—C2	−75.4 (6)	C4—C5—C6—N6	179.4 (7)
N1—N2—C3—N3	176.6 (6)	C2—C5—C6—N6	−1.5 (13)
Cs1^vii^—N2—C3—N3	−28.5 (7)	O3—N6—C6—N5	9.6 (11)
Cs2^ix^—N2—C3—N3	101.4 (6)	O4—N6—C6—N5	−171.2 (7)
C1—C2—C3—N2	0.7 (7)	O3—N6—C6—C5	−170.2 (8)
C5—C2—C3—N2	−177.8 (6)	O4—N6—C6—C5	9.0 (11)

Symmetry codes: (i) −*x*+1, *y*−1/2, −*z*+2; (ii) −*x*, *y*−1/2, −*z*+1; (iii) *x*, *y*−1, *z*; (iv) *x*+1, *y*, *z*; (v) *x*, *y*, *z*−1; (vi) −*x*, *y*+1/2, −*z*+1; (vii) −*x*+1, *y*+1/2, −*z*+2; (viii) *x*, *y*+1, *z*; (ix) *x*, *y*, *z*+1; (x) *x*−1, *y*, *z*.

### Poly[[[µ-3,3'-dinitro-4,4'-bipyrazole-1,1'-diido]dicaesium] monohydrate] (3) Hydrogen-bond geometry (Å, º)

**Table:**

*D*—H···*A*	*D*—H	H···*A*	*D*···*A*	*D*—H···*A*
O1*W*—H1*W*···N1^vii^	0.85	2.01	2.855 (7)	171
O1*W*—H2*W*···N5^vi^	0.85	2.50	3.334 (9)	168
C1—H1···O2^iii^	0.94	2.83	3.508 (10)	130
C4—H4···O1^xi^	0.94	2.62	3.547 (9)	168

Symmetry codes: (iii) *x*, *y*−1, *z*; (vi) −*x*, *y*+1/2, −*z*+1; (vii) −*x*+1, *y*+1/2, −*z*+2; (xi) −*x*, *y*−1/2, −*z*+2.
